# The cellular phenotype of cytoplasmic incompatibility in *Culex pipiens* in the light of *cidB* diversity

**DOI:** 10.1371/journal.ppat.1007364

**Published:** 2018-10-15

**Authors:** Manon Bonneau, Frédéric Landmann, Pierrick Labbé, Fabienne Justy, Mylène Weill, Mathieu Sicard

**Affiliations:** 1 ISEM, University of Montpellier, CNRS, EPHE, IRD, Montpellier, France; 2 CRBM, University of Montpellier, CNRS, Montpellier, France; Pennsylvania State University, UNITED STATES

## Abstract

*Wolbachia* are maternally inherited endosymbiotic bacteria, widespread among arthropods thanks to host reproductive manipulations that increase their prevalence into host populations. The most commonly observed manipulation is cytoplasmic incompatibility (CI). CI leads to embryonic death in crosses between i) infected males and uninfected females and ii) individuals infected with incompatible *Wolbachia* strains. CI can be conceptualized as a toxin-antidote system where a toxin deposited by *Wolbachia* in the sperm would induce embryonic death unless countered by an antidote produced by *Wolbachia* present in the eggs. In *Drosophila melanogaster*, transgenic expression of *Wolbachia* effector *cidB* revealed its function of CI-inducing toxin. Moreover in *Culex pipiens*, the diversity of *cidB* variants present in *w*Pip strains accounts for the diversity in crossing-types. We conducted cytological analyses to determine the CI mechanisms that lead to embryonic death in *C*. *pipiens*, and assess whether diversity in crossing-types could be based on variations in these mechanisms. We revealed that paternal chromatin condensation and segregation defects during the first embryonic division are always responsible for embryonic death. The strongest observed defects lead to an exclusion of the paternal chromatin from the first zygotic division, resulting in haploid embryos unable to hatch. The proportion of unhatched haploid embryos, developing with only maternal chromatin, which reflects the frequency of strong defects can be considered as a proxy of CI intensity at the cellular level. We thus studied the putative effect of variations in crossing types and *cidB* diversification on CI defects intensity. Incompatible crosses involving distinct *w*Pip strains revealed that CI defects intensity depends on the *Wolbachia* strains hosted by the males and is linked to the diversity of *cidB* genes harbored in their genomes. These results support that, additionally to its implication in *C*. *pipiens* crossing type variability, *cidB* diversification also influences the strength of CI embryonic defects.

## Author summary

In some crosses, mosquito males belonging to the species *Culex pipiens* prevent their females from having live progenies. This phenomenon called cytoplasmic incompatibility (CI) is caused by intracellular bacteria named *Wolbachia*. CI occurs when males infected with *Wolbachia* fertilize females infected with genetically distinct incompatible *Wolbachia* resulting in the death of all the embryos. At the world scale, crossing relationships between *C*. *pipiens* are quite puzzling. Despite this complexity in crossing relationships and the diversity of *cidB* genes involved in CI mechanisms in *C*. *pipiens*, we demonstrate a single shared CI cellular phenotype leading to the death of the embryos: the paternal chromatin exclusion from the first embryonic division. If paternal chromatin is fully excluded, embryos developed with haploid set of chromosomes. We show that the frequency of haploid development varies according to the *Wolbachia* strains hosted by the males which differ in the *cidB* variants harbored in their genomes. Absence of *Wolbachia* in the eggs totally block haploid development showing that maternal *Wolbachia* presence interplays with CI mechanisms in a way that allows haploid development to occur. Understanding CI mechanism in mosquitoes is the corner stone to build new sustainable and adaptable *Wolbachia* based strategies for vector control.

## Introduction

*Wolbachia* are maternally-inherited endosymbionts, widespread among arthropods and filarial nematodes [1,2], and the most frequent endocytobiotic bacteria detected in arthropods [3]. This high prevalence is attributed to their ability to manipulate their host reproduction to spread within arthropod populations [1]. The main reproductive manipulation strategy used by *Wolbachia* is named cytoplasmic incompatibility (CI) [4]. CI is a form of conditional sterility resulting in embryonic lethality [5]. In most of the host species, CI occurs when males infected with *Wolbachia* fertilize uninfected females whereas the reciprocal cross remains compatible. This difference in the production of viable offspring between infected and uninfected female reproduction enhances the spread of *Wolbachia* in host’s populations [6]. CI can also occur between males and females both infected with different and incompatible *Wolbachia* strains [7–12]. In such situations, incompatibility can be either unidirectional (one cross direction is compatible while the reciprocal one is incompatible) or bidirectional (both cross directions are incompatible) [7–9]. The penetrance of CI, *i*.*e*. the number of embryos affected by CI in a cross, varies depending on the *Wolbachia* strain and the host involved in the interaction. Indeed, in the same host *Drosophila simulans*, *w*Ri induces complete CI (*i*.*e*. crosses in which all the embryos are affected by CI), while *w*No and *w*Ha strains induce lower levels of CI, *i*.*e*. some embryos can develop [13]. Complete CI penetrance was also described in *Nasonia spp*. depending on the *Wolbachia* strain involved and in all *Culex pipiens* incompatible crosses [14,15]. The variability of CI penetrance has been correlated to different factors such as the *Wolbachia* density in the sperm and eggs [16,17], host age [18] and host nuclear genotype [19].

Cellular consequences of *Wolbachia*-induced CI have been monitored during embryogenesis in *D*. *simulans*, *D*. *melanogaster* and *N*. *vitripenis* [5,20–23]. These studies revealed common cellular defects in these three species: a delay in paternal chromatin condensation and segregation defect during the first mitotic division of the embryo [23–25]. In *D*. *melanogaster*, a delay in histone H3.3 deposition after protamine removal on the paternal chromatin was observed and linked to chromatin remodeling defects [22]. This remodeling defect was associated with the persistence of the DNA replication factor PCNA (Proliferating Cell Nuclear Antigen) during mitosis, reflecting incomplete replication of paternal DNA [22]. It has been envisioned that these DNA replication defects might be responsible for the paternal chromatin bridges and segregation failure during the first mitotic division, which result in early embryonic arrest [22,24,25]. Nevertheless, some embryos reaching late development stages have been reported in CI crosses [21,25,26]. Late development in CI embryos was interpreted as resulting from a complete paternal chromatin exclusion during the first division, which allows successful maternal chromatin segregation and the formation of two haploid nuclei [25]. These haploid nuclei which further divide, lead to gynogenetic development (*i*.*e*. haploid development with only maternal genetic material) until late embryonic stages [27]. These haploid embryos are never viable in diploid species such as *D*. *simulans* [25]. However, in the haplodiploid parasitoid wasps *Leptopilina heterotoma* and *N*. *vitripenis*, CI*-*induced paternal chromosome defects can lead either i) to the death of the embryos or ii) to the production of healthy males [19,21,28,29]. It has been proposed that these two CI developmental outcomes could result from different degree of paternal chromatin defect (improper condensation) before the first division [29–31]. Severe defects would lead to complete elimination of male chromosomes from the first zygotic division resulting in haploidization and male development, whereas less severe defects would lead in partial exclusion of the paternal chromatin resulting in incomplete elimination of male chromosomes and early arrest of the aneuploid development [29–31]. In diploid species such as *C*. *pipiens* and *D*. *melanogaster*, the proportion of unhatched developed haploid embryos observed in fully incompatible CI crosses would be a proxy of the frequency of total paternal chromatin exclusion during embryogenesis due to strong CI intensity at the cellular level. However, this hypothesis is counter intuitive as one could expect that strong CI defects would prevent any development to occur while soft CI defect would allow development.

The molecular mechanism underlying CI can be conceptualized as a toxin-antidote system in which i) a toxin produced by *Wolbachia* in the testes, more generally called a “*mod* factor”, and introduced in the sperm during spermatogenesis would interfere (“modify”) with the paternal chromatin and induce embryonic perturbations, and ii) an antidote released by *Wolbachia* in the egg, more generally called “*resc* factor”, would “rescue” these paternal chromatin defects to allow normal embryogenesis to occur [32,33]. The recent discoveries of i) *Wolbachia* genes *cidA* and *cidB* ability to recapitulate the CI phenotypes when expressed in transgenic *Drosophila* [34,35], and ii) the link between specific allelic *cidA*^*w*Pip^*/cidB*^*w*Pip^ variations in worldwide natural *C*. *pipiens* populations and the capacity of males to sterilize females [36], open new paths into understanding CI mechanisms. *CidA* and *cidB* genes are syntenic genes within the WO phage region (S6 Table) [34,35,37–39]. *CidB* encodes a deubiquitylating enzyme (DUB) and when a *cidB*^*w*Pip^ construct bearing this catalytically inactivated DUB domain was expressed in *D*. *melanogaster* males, CI was no longer observed, showing the implication of the deubiquitylating activity in the *mod* function [34]. The role of *cidA* in the CI mechanism is more debated because i) both *cidA*^*w*Mel^ and *cidB*^*w*Mel^ are required to induce CI in transgenic *Drosophila* [35] and ii) in natural populations of *C*. *pipiens*, specific *cidA*^*w*Pip^ allelic variations were found to be linked to *mod* variations [36]. However, the implication of *cidA* in the *resc* function is supported by the capacity of *cidA* to prevent *cidB* toxicity in yeast [34] and the capability of transgenic uninfected females expressing *cidA*^*w*Mel^ throughout oogenesis to rescue the effect of *cidB*^*w*Mel^ [40].

In *C*. *pipiens*, all individuals are infected with different *Wolbachia* strains belonging to the monophyletic *w*Pip group, but divided in five subgroups *w*PipI to *w*PipV. MLST (Multi Locus Sequence Typing) genes from Baldo et al. (2006) [41] were not polymorphic between *w*Pip strains, thus a *w*Pip specific MLST with more polymorphic genes MutL, ank2, pk1, pk2, GP12, GP15, and RepA was used to resolve *w*Pip phylogeny (S6 Table) [12]. Mosquitoes hosting *w*Pip from the same group are likely to be compatible with each other but incompatible with mosquitoes infected with *Wolbachia* from other *w*Pip groups [42]. This diversity of *w*Pip strains distributed all around the world is responsible for the unique complexity of CI crossing types described in this host species [12,43]. Unlike *w*VitA and *w*Mel, which harbors only one *cidA*/*cidB* copy, and *w*Ri, which harbors two identical copies of *cidA*/*cidB*, high intra and inter-genomic diversities of *cidA*^*w*Pip^*/cidB*^*w*Pip^ genes were uncovered between and within all *w*Pip strains studied [36]. This diversity certainly explains the unrivaled diversity of crossing types described in *C*. *pipiens* [36]. This *cidA*^*w*Pip^*/cidB*^*w*Pip^ genes amplification and diversification within the same *Wolbachia* genome may also account for the impressive CI penetrance described in *C*. *pipiens*. Indeed, expression of multiple *cidA*^*w*Pip^ and *cidB*^*w*Pip^ variants in males could i) be responsible for differences in CI cellular phenotype(s) and ii) influence the penetrance of CI. Here, we investigated the putative impact of crossing type variations and *cidA*^*w*Pip^*/cidB*^*w*Pip^ diversification on CI cellular phenotypes and CI intensity during *C*. *pipiens* embryogenesis. To this end, we monitored the development of embryos derived from various incompatible crosses involving males from *C*. *pipiens* lines infected with *Wolbachia* strains from distinct *w*Pip groups and exhibiting different crossing types.

## Results

### A single cellular phenotype of CI in *C*. *pipiens*

Three different types of crosses were performed using different laboratory mosquito lines: i) fertile crosses between individuals from the same line, representing our control to monitor normal embryonic early development, ii) sterile crosses between mosquito lines harboring different *w*Pip strains, and iii) sterile crosses between infected males and uninfected females (TC lines), to test the effect of *Wolbachia* absence on embryo development and CI cellular mechanism (S1 and S2 Tables).

**Fig 1:**
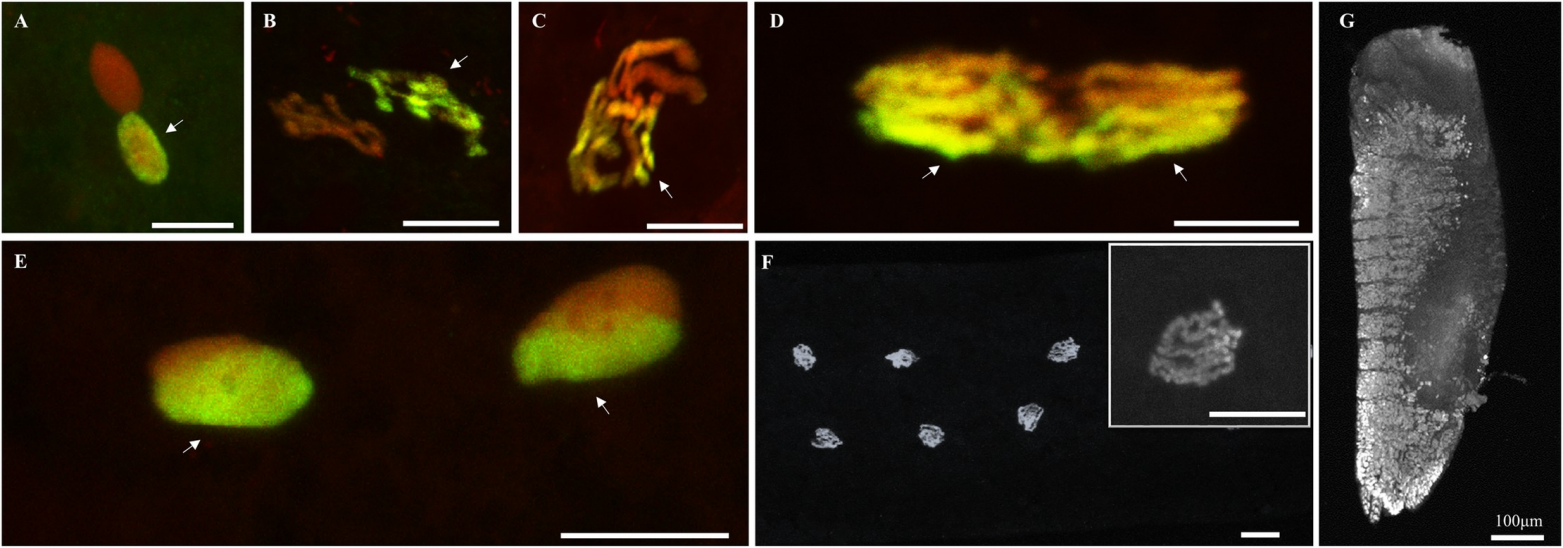
Normal embryogenesis in *C*. *pipiens*: From first nuclear divisions to segmentation. Paternal chromatin appears in green/yellow (acetylated histone H4 labelling is dominant) and maternal chromatin appears in red (propidium iodide labelling is dominant). (A) apposition of maternal and paternal pronuclei, (B) chromatin under condensation, (C) condensed chromatin, (D) first mitotic division anaphase (maternal and paternal chromosome segregate independently), (E) two nuclei following the first division, (F) normal diploid development 1 hour after oviposition, six diploid nuclei are visible after 1h development, (G) segmented embryo after 24 hours of development. White arrows indicate the paternal chromatin. Confocal stacks were obtained on embryos from several fertile intra-line crosses due to the difficulty to obtain all the early embryonic stages from each cross (S2 Table). Scale bar is 10μm.

The cellular phenotype during embryogenesis in fertile intra-line crosses is illustrated in Fig 1. To differentially visualize the paternal from the maternal chromatin, we used propidium iodide to mark both maternal and paternal chromatin and an anti-acetylated histone H4 labelling that preferentially marks the *de novo* assembled paternal chromatin after protamine removal [22]. Paternal chromatin appears in green/yellow (acetylated histone H4 labelling is dominant) and maternal chromatin appears in red (propidium iodide labelling is dominant). After fertilization, maternal and paternal pronuclei migrated toward each other and apposed (documented embryos with confocal microscopy images n = 4, Fig 1A). Then, paternal and maternal chromatins condensed and entered into first mitotic division (n = 3, Fig 1B). During the first division, paternal and maternal chromosomes aligned in separate region at the metaphase plate (n = 1, Fig 1C). Both sets of chromosomes segregated equally during anaphase (n = 3, Fig 1D) to produce two diploid nuclei (n = 1, Fig 1E) that proliferate mitotically (n = 16, Fig 1F). After 24 hours of development, organogenesis was ongoing and segmentation was clearly visible (n = 2, Fig 1G).

**Fig 2:**
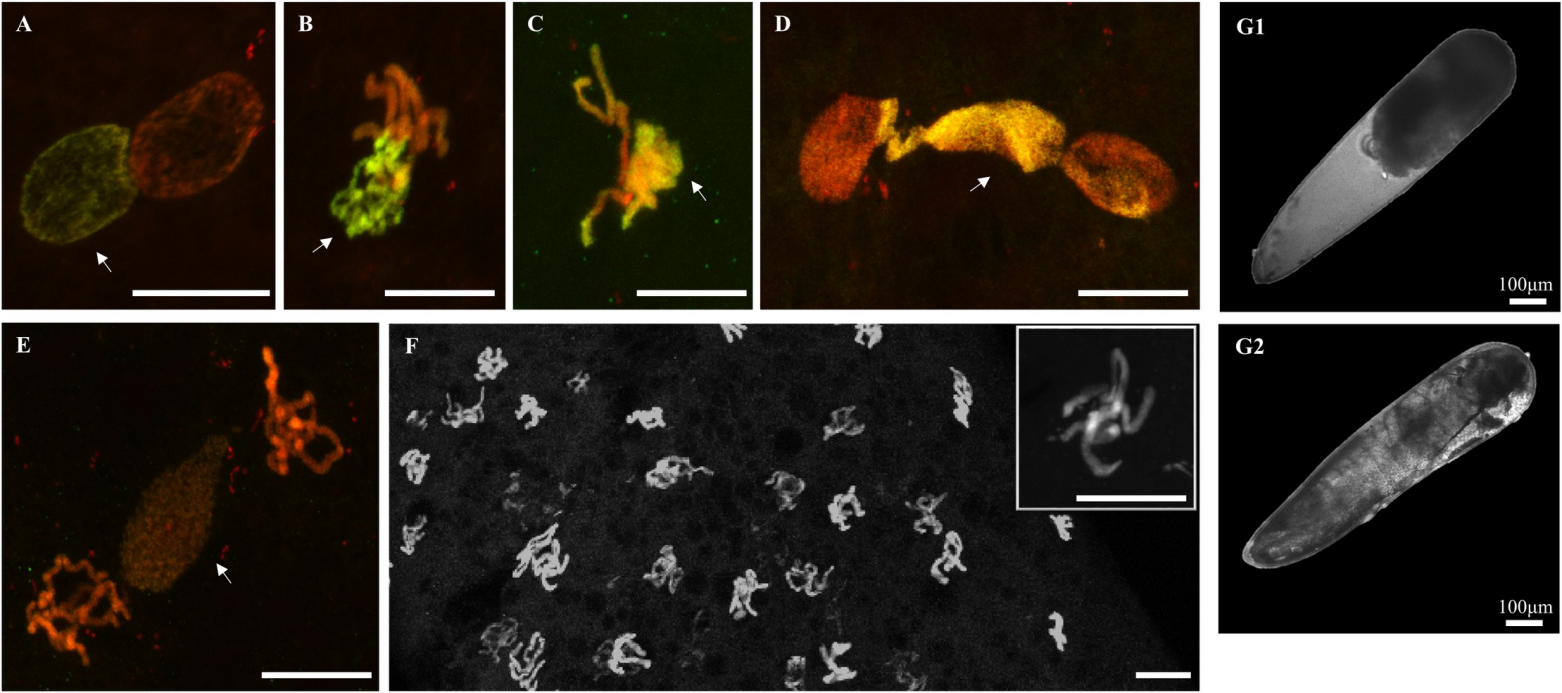
CI embryos from incompatible infected parents in *C*. *pipiens*: From first nuclear divisions to segmentation. Paternal chromatin appears in green/yellow (acetylated histone H4 labelling is dominant) and maternal chromatin appears in red (propidium iodide labelling is dominant) (A) apposition of maternal and paternal pronuclei, (B) delay in paternal chromatin condensation, (C) condensed maternal chromatin and under-condensed paternal chromatin, (D) paternal chromatin failed to segregate and form a chromatin bridge between segregating maternal chromatin, (E) two nuclei containing mainly maternal chromatin while paternal chromatin do not segregate, (F) haploid development 2 hours after oviposition, (G) the two possible fates of development after 48 hours (1) non-viable embryo with no visible development, and (2) unhatched developed embryo with visible segments. White arrows indicate the paternal chromatin. Confocal stacks (panels A,B,C,D,E,F) and optical images (panels G1 and G2) were obtained on embryos from several CI crosses between infected males and females due to the difficulty to obtain all the early embryonic stages for each cross (S2 Table). Green dots are background noises likely due to the presence of residual antibodies. Scale bar is 10μm.

**Fig 3:**
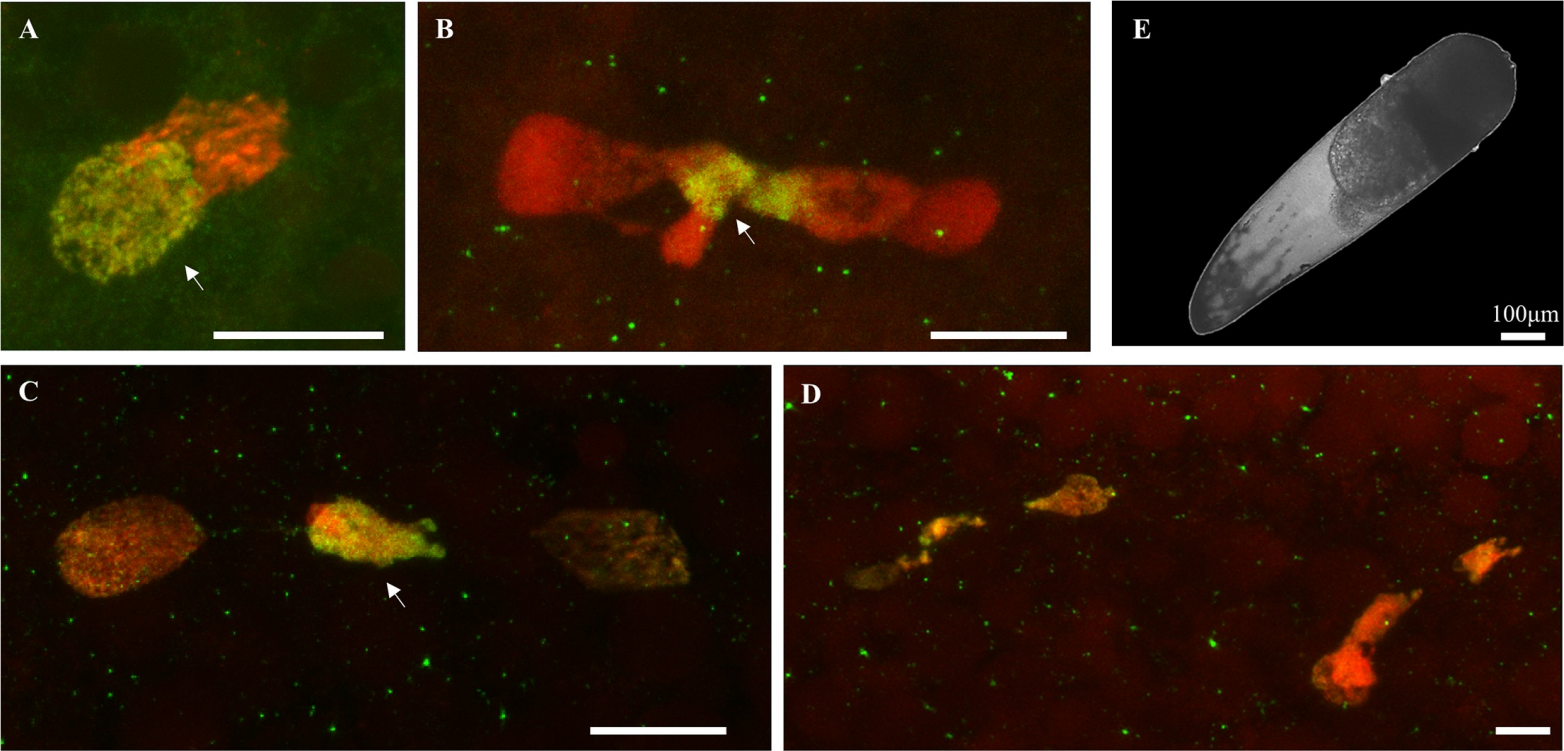
CI embryos from infected fathers and uninfected mothers: An arrest after the first nuclear divisions. Paternal chromatin appears in green/yellow (acetylated histone H4 labelling is dominant) and maternal chromatin appears in red (propidium iodide labelling is dominant) (A) apposition of maternal and paternal pronuclei, (B) paternal chromatin failed to segregate and form a chromatin bridge between segregated maternal chromatin, (C) two nuclei containing mainly maternal chromatin while paternal chromatin do not segregate, (D) abnormal development at 2 hours after oviposition: only few aborted divisions are observed, (E) none of the embryos from such crosses exhibited any visible development under microscope 48 hours post-oviposition. White arrows indicate the paternal chromatin. Confocal stacks (panels A,B,C,D,) and optical images (panels E) were obtained on embryos from several CI crosses between infected males and uninfected females due to the difficulty to obtain all the early embryonic stages for each cross (S2 Table). Green dots are background noises likely due to the presence of residual antibodies. Scale bar is 10μm.

In sterile crosses between two infected incompatible *C*. *pipiens* lines (Fig 2) as well as in crosses between infected males and uninfected females (Fig 3), paternal and maternal pronuclei migrated and apposed normally (n = 2, Figs 2A and 3A). However, during the early prophase, paternal chromatin appeared under-condensed compared to maternal chromatin (n = 2, Fig 2B and 2C). Then the paternal chromatin failed to segregate properly during anaphase (n = 16, Figs 2D, 2E, 3B and 3C). In telophase paternal chromatin can either i) form chromatin bridges between the two maternal nuclei (n = 10, Figs 2D and 3B), certainly causing the early arrest of embryogenesis and production of undeveloped embryos (Figs 2G1 and 3E), or ii) appear fully excluded (n = 6, Figs 2E and 3C), allowing maternal chromatin to successfully segregate and eventually formed unhatched haploid developed embryos presenting eyes and segments (Fig 2G2).

Eight distinct CI crosses were monitored between males and females infected with *w*Pip strains belonging to different *w*Pip groups, and two distinct CI crosses were monitored between infected males and uninfected females (S2 Table). However, despite this diversity of CI crosses, condensation and segregation defects of the paternal chromatin were the only observed cellular defects resulting in embryonic death, and were never observed in any embryo resulting from fertile crosses (seven intra-line fertile crosses, S2 Table).

### Absence of *Wolbachia* in the oocytes blocks embryogenesis in CI embryos

2 hours after oviposition, some embryos resulting from CI crosses between infected lines pursued their embryogenesis (n = 2, Fig 2F), and after 48 hours these embryos exhibited visible development, as segmentation was clearly observable under optical microscope (Fig 2G2). However, more than 99.9% of these developed embryos did not hatch. In the sterile cross between ♂ Slab x ♀ Ichkeul 13, unhatched developed embryos only displayed maternal markers (see Material and methods, "Ploidy determination in CI developed embryos", S1 Fig), showing that they were composed of only haploid maternal DNA, as previously described in Duron and Weill (2006)[44].

All the seven different crosses performed between males infected with different *w*Pip strains and uninfected females from different TC-treated lines produced 100% of non-developed embryos (Fig 3E and S3 Table). Confocal observations of these embryos showed that only few and abnormal nuclei were observed in the cytoplasm 2 hours post oviposition (n = 5, Fig 3D), indicating an early arrest of the embryogenesis (S2 Table).

### Males infected by different *w*Pip with distinct *mod* profiles induced different CI defects intensities

It has been previously proposed that the production of haploid or aneuploid embryos in CI crosses represented a proxy of intensity of CI defects that leads to more or less complete paternal chromatin exclusion [29–31,44]. Severe defects would lead to the complete exclusion of the paternal chromatin during the first embryonic division (*i*.*e*. strong cellular CI intensity), which would allow maternal chromatin successful segregation and the production of a developed haploid embryo. Thus, unhatched developed haploid embryos reflect the occurrence during the first zygotic division of strong CI defects while unhatched non-developed embryos illustrate the occurrence of weak CI defects. We used this link between the degree of paternal chromatin exclusion (*i*.*e*. weak or strong cellular CI) and the proportion of unhatched developed embryos in eggs-rafts from incompatible crosses to investigate the variability of CI intensity (*i*.*e*. frequency of strong *versus* weak CI defects).

**Fig 4:**
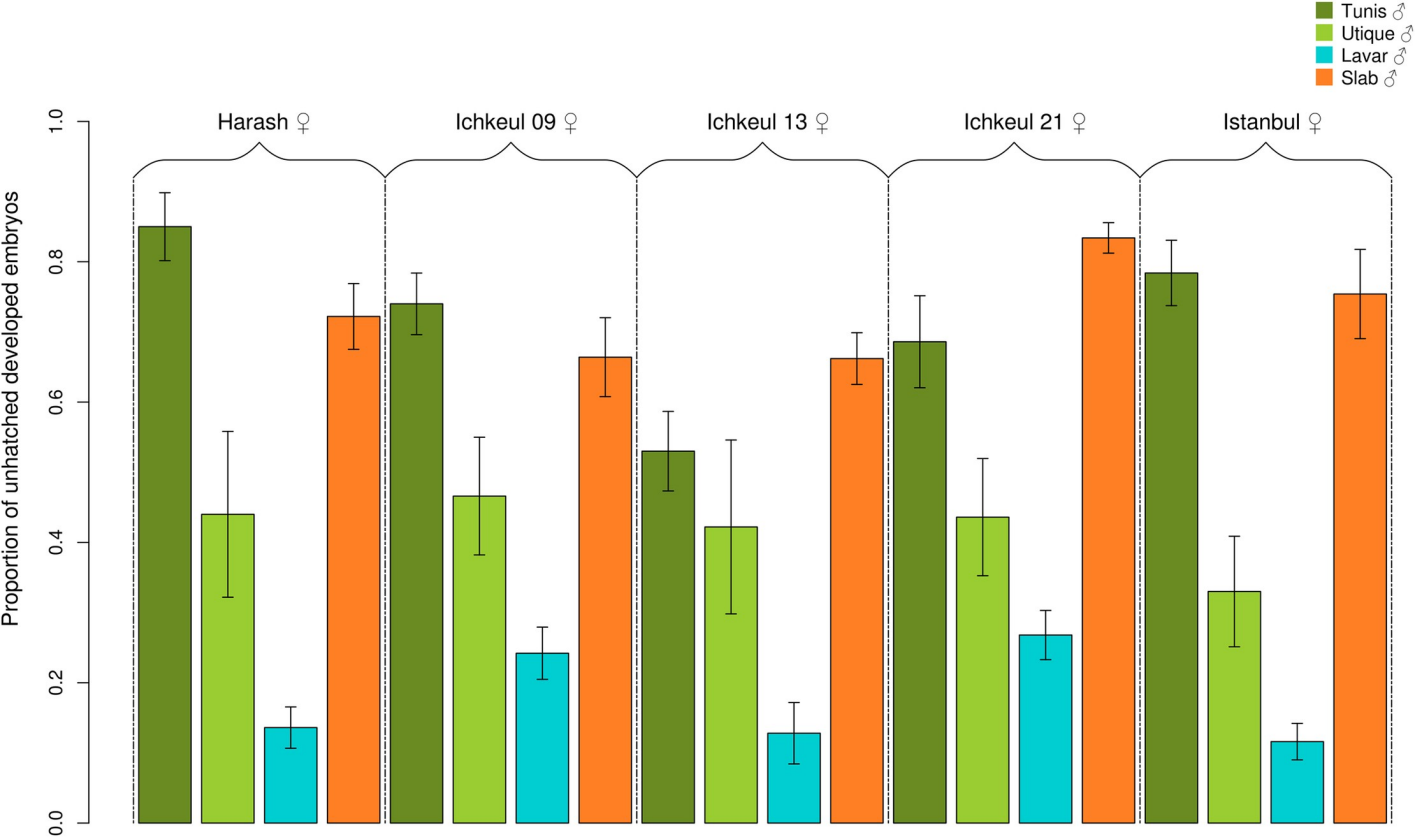
Variations in cellular CI intensity. Infected males from four lines exhibiting different *mod* profiles (Mal lines) and females from five lines with the same *resc* profile (Fem lines) were crossed and resulted in the 20 studied CI crosses (*i*.*e*. >99,9% of the embryos died before hatching). The vertical bars represent the proportion of unhatched developed embryos in each of these crosses, the four different colors represent the four different Mal lines, each group of four bars corresponding to the same Fem line. Error bars represent the standard error. The proportion of unhatched developed embryos was significantly different between crosses involving males infected with distinct *w*Pip responsible for different *mod*; no significant effect of the Fem lines was detected.

Using this proxy, we studied the variation in CI intensity between 20 incompatible crosses between infected lines (S3 Table). These 20 crosses involved i) males from four different isofemale lines (Mal lines) infected with *w*Pip strains from different *w*Pip groups all exhibiting distinct *mod* profiles, and ii) females from five isofemale lines (Fem lines) all harbouring *w*Pip strains from the *w*PipIV group and exhibiting the same *resc* profile [36,42] (S4 Table). Significant differences were found regarding the proportion of unhatched developed embryos between these incompatible crosses (generalized linear model (GLM), χ ^2^ = 245.695, df = 19, p< 0.001, Fig 4, Table 1 and S3 Table). While no effect of Fem lines was detected on this proportion (GLMM, χ^2^ = 2.508, df = 4, p = 0.643, Fig 4), the Mal lines involved in the crosses had a significant effect (GLMM, χ^2^ = 16.211, df = 3, p = 0.001, Table 1 and Fig 4). Males from Tunis (*w*PipI *mod* ii) and Slab (*w*PipIII *mod* iii) lines induced the highest proportion of developed embryos (72% and 73%, respectively) but were not significantly different from one another (GLMM, χ^2^ = 0.002, df = 1, p = 0.968); males from Utique (*w*PipI *mod* iv) and Lavar (*w*PipII *mod* vi) lines induced significantly different and lower proportions of unhatched developed embryos (respectively 42% and 18%, Table 1). The nuclear genetic background of the males seems not to be involved in the variability of CI defects intensity: males from backcrossed line Sl(*w*PipI-Tunis) and males from the Tunis line, which host the same *w*PipI strain in different genetic backgrounds, indeed induced similar unhatched developed embryos proportions when crossed with the five Fem lines (0.71 ± 0.22 and 0.72 ± 0.19 respectively; GLMM, χ^2^ = 0.008, df = 1, p = 0.927). Consequently variability in CI defects intensity appears to be only dictated by the *w*Pip strain harbored by the different males.

**Table 1:** Males with different *mod* profiles: Proportions of unhatched developed embryos, *cidA*-*cidB* copy number and expression.

Line	Proportion of unhatched developed embryo	*cidA* copy number	*cidB* copy number	*cidA/cidB* copy number	*cidA* expression	*cidB* expression	*cidA/cidB* expression
*w*PipI-Tunis	0.72 ± 0.19* (a)	5.98 ± 0.73 (a)	5.57 ± 0.56 (a)	1.08 ± 0.14 (a)	0.75 ± 0.18 (a)	0.50 ± 0.16 (a)	1.55 ± 0.30 (a)
*w*PipI-Utique	0.42 ± 0.31 (b)	5.02 ± 0.41 (b)	4.76 ± 1.00 (b)	1.08 ± 0.16 (a)	0.76 ± 0.27 (a)	0.49 ± 0.13 (a)	1.53 ± 0.32 (a)
*w*PipII-Lavar	0.18 ± 0.12 (c)	4.98 ± 0.83 (b)	4.14 ± 0.76 (c)	1.22 ± 0.22 (a)	0.97 ± 0.24 (a)	0.47 ± 0.15 (a)	2.14 ± 0.58 (b)
*w*PipIII-Slab	0.73 ± 0.16 (a)	4.69 ± 0.42 (b)	4.07 ± 0.47 (c)	1.17 ± 0.18 (a)	0.94 ± 0.26 (a)	0.60 ± 0.20 (a)	1.62 ± 0.32 (a)

*The average proportion of unhatched developed embryos, the number of copies of *cidA* and *cidB* and their ratio, as well as the expression levels of *cidA* and *cidB* and their ratio are indicated as the Mal line means ± standard deviations.

a, b, c letters represent statistical groups (*i*.*e*. means with the same letter are not significantly different).

### Genetic investigations of cellular CI intensity variation

The results from the previous section indicate that the proportion of unhatched developed embryos in CI crosses likely depended on variations in the males' *mod* profiles. To investigate the sources of such variation in CI defects, we tested the putative influence of several variables: i) the density of *Wolbachia* in the testes, ii) the copy numbers of *cidA* and *cidB* genes in the different *w*Pip genomes, iii) the expression levels of *cidA* and *cidB*, and iv) the *cidA* and *cidB* variants repertoires in the genomes of the different *w*Pip strains hosted by the males.

#### *Wolbachia* density was significantly lower in Lavar males' testes

**Fig 5:**
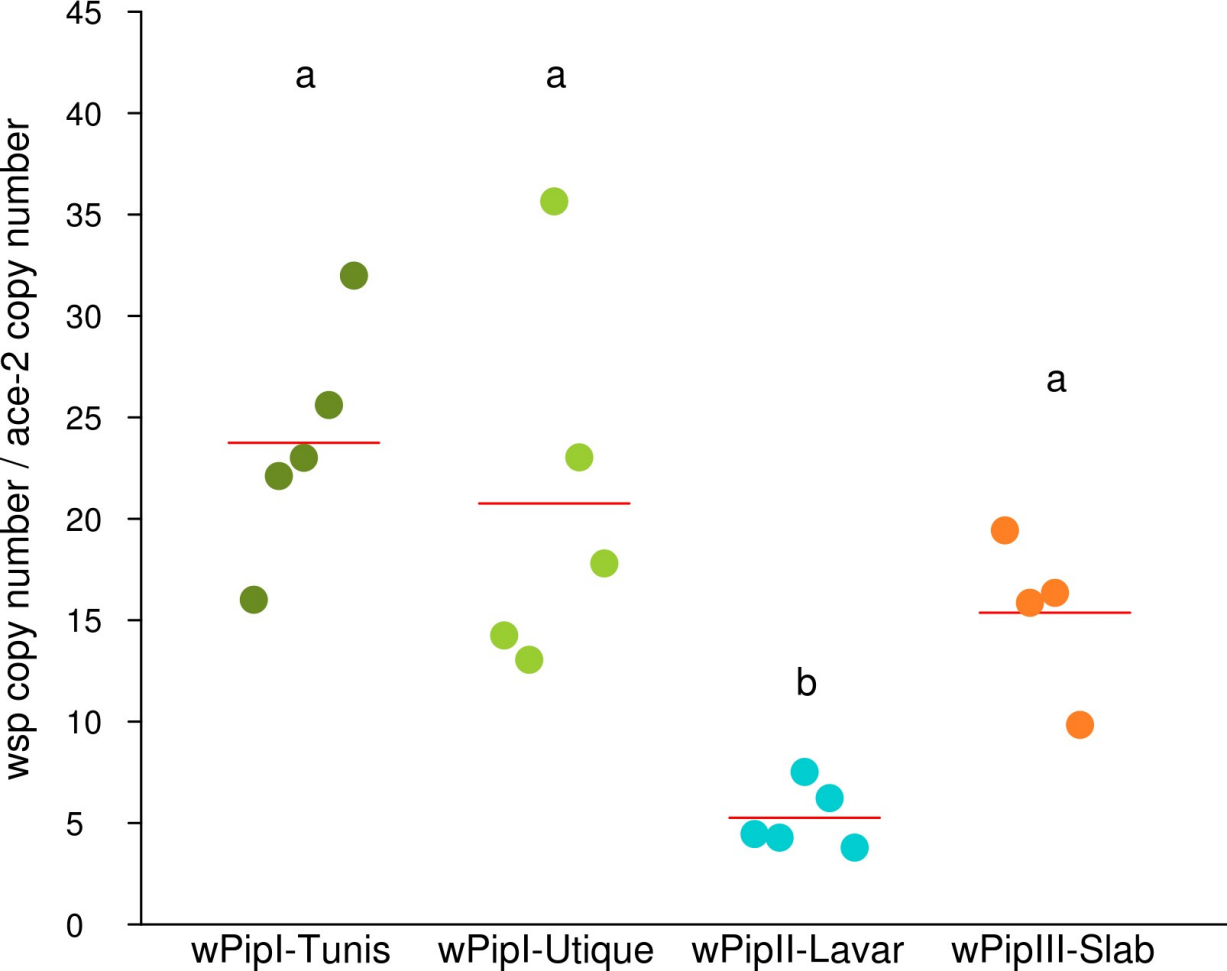
*Wolbachia* density in testes. *Wolbachia* densities in mosquito testes were measured by quantitative PCR as the ratio between the number of copies of the *Wolbachia wsp* gene and the *C*. *pipiens ace-2* nuclear gene. The colored dots represent the average density of *Wolbachia* in a pool of three pairs of testes and the red strips represent the average *Wolbachia* density for each line/*w*Pip strain. Letters represent the different statistical groups (*i*.*e*. means with the same letter are not significantly different), showing that Lavar males harbored significantly less *Wolbachia* in their testes than the other males.

Testicular *Wolbachia* densities were not significantly different between males from Tunis, Utique and Slab lines (GLM, F = 3.919, df = 1, p = 0.065), but significantly lower in Lavar males (GLM, F = 9.337, df = 3, p<0.001, Fig 5).

#### *CidA* and *cidB* copy numbers differed between the *w*Pip strains

Number of genomic copies of *cidA* per *w*Pip strain (*i*.*e*. *Wolbachia* cells from the same *C*. *pipiens* line) varied between Mal lines from 4.68 ± 0.42 to 5.98 ± 0.73 copies, *w*PipI-Tunis displaying a significantly higher *cidA* copy number than the other strains (GLM, F = 8.077, df = 3, p<0.001, Table 1, S5 Fig). Number of genomic copies of *cidB* also varied from 4.07 ± 0.47 to 5.57 ± 0.56 copies (GLM, F = 9.142, df = 3, p<0.001), and was found significantly higher in *w*PipI-Tunis than in *w*PipI-Utique, *w*PipIII-Slab and *w*PipII-Lavar displaying significantly lower but similar copy numbers (Table 1, S6 Fig). Despite these differences, the *cidA/cidB* copy number ratios were not significantly different and close to one, for all *w*Pip strains (GLM, F = 1.504, df = 3, p = 0.230, Table 1, S7 Fig).

#### Variation in *cidA* and *cidB* relative expression levels

The expression levels of both *cidA* and *cidB* were not different between the four *C*. *pipiens* Mal lines (GLM, *cidA*: F = 2.409, df = 3, p = 0.083, *cidB*: F = 1.239, df = 3, p = 0.310, Table 1, S8 and S9 Figs). *CidA* was found about 1.5 times more expressed than *cidB*, except for *w*PipII-Lavar which *cidA*/*cidB* expression level ratio appeared significantly higher (GLM, F = 5.447, df = 3, p = 0.003, Table 1, S10 Fig).

#### *CidA* and *cidB* variants repertoires were variable between *w*Pip strains

Cloning and Sanger sequencing revealed that the four Mal lines with different *mod* were infected with *Wolbachia* strains that harbored different *cidA* and *cidB* variant repertoires (S2 and S3 Figs). No *cidA* or *cidB* nucleotide sequence variant were shared between the three *w*Pip groups. However, the CidA_II(α/1) variant of *w*PipII-Lavar and the CidA_III(β/8) variant of *w*PipIII-Slab presented the same amino-acid sequence (S2 Fig). *w*PipIII-Slab exhibited ten variants of *cidA* based on their nucleotide sequences (however only seven of them differed in their amino-acid sequences), *w*PipII-Lavar three, *w*PipI-Tunis four and *w*PipI-Utique seven (S2 Fig). Both *w*PipII-Lavar and *w*PipI-Utique carried two different variants of *cidB*, while *w*PipI-Tunis and *w*PipIII-Slab carried four (S3 Fig).

### Correlations between CI defects intensity and *w*Pip genetic variations

Correlative analyses were conducted to assess the potential links between variations in CI defects intensity and genetic variations. We found no significant correlations between the proportion of unhatched developed embryos in CI crosses and i) *Wolbachia* density in the testes (Spearman, ρ = 0.4, p = 0.750), ii) *cidA* copy number (Spearman, ρ = -0.2, p = 0.917), iii) *cidB* copy number (Spearman, ρ = -0.2, p = 0.917), iv) *cidA*/*cidB* copy number ratio (Spearman, ρ = -0.4, p = 0.750), v) *cidA* expression levels (Spearman, ρ = -0.4, p = 0.750), vi) *cidB* expression levels (Spearman, ρ = 1, p = 0.083), vii) *cidA* over *cidB* expression levels (Spearman, ρ = -0.2, p = 0.917) and viii) the number of different *cidA* variants in the repertories (Spearman, ρ = 0.8, p = 0.333). However, males infected with *w*Pip strains with 4 *cidB* variants induced significantly higher proportions of unhatched developed embryos (*w*PipI-Tunis and *w*PipIII-Slab mean: 0.72 ± 0.17) than males infected with *w*Pip strains with only 2 *cidB* variants (*w*PipII-Lavar and *w*PipI-Utique; mean: 0.30 ± 0.26, Wilcoxon, W = 1159, p<0.001, S4 Fig).

## Discussion

To investigate whether the high diversity of *cidA*/*cidB* variants within *w*Pip could be responsible for variations in the cellular phenotype of CI, we studied the development of *C*. *pipiens* embryos resulting from various incompatible crosses. The early embryogenesis was assessed using fluorescence confocal microscopy in i) fertile intra-line crosses, ii) incompatible crosses between infected males and infected females, and iii) incompatible crosses between infected males and uninfected females. Despite the diversity of performed crosses between males and females infected with *w*Pip strains harboring different *cidA*/*cidB* variants repertoires or uninfected female, a unique and recurrent embryonic phenotype was detected, consisting in paternal chromatin condensation and segregation defects during the first embryonic division (Figs 2B, 2E, 3B and 3C). This phenotype was never detected in any embryos derived from intra-line crosses (Fig 1). Hence the diversity of *cidA*/*cidB* variants repertoires describes in *C*. *pipiens* does not seem to influence the CI mechanism itself, which is consistent with all CidB variants carrying a conserved DUB domain [36]. Similar defects were already reported in both *Drosophila* and *Nasonia* [23,25], suggesting an universality of *Cid* induced-cellular CI mechanism whenever *cid* genes are diversified or not in the *Wolbachia* genome.

An unsolved question is the molecular pathway(s) targeted by CidA and CidB. Most protein domains within CidA and CidB remain to be characterized and how they interact with each other and host targets to induce CI remains unclear. However, a first tangible element is that the catalytically active DUB domain (involved in deubiquitination) in CidB proteins, which is considered as involved in the *mod* function, is necessary to induce CI in transgenic *Drosophila* [34]. Ubiquitination pathways have been shown to be crucial for many essential cellular processes, such as the regulation of the chromatin dynamics and the cell cycle progression [45]. Changes in ubiquitination could for instance directly or indirectly affect H3.3 histone incorporation after protamine removal and DNA replication as suggested by PCNA persistence on the paternal chromatin [22], which would result in an asynchronous mitotic entry of paternal and maternal pronuclear chromatin [30]. Interestingly, *Cardinium*, an endosymbiont phylogenetically distant from *Wolbachia*, induces CI with quite similar embryogenesis defects in the hymenoptera *Encarsia suzanna* [46]. Moreover, an ubiquitin specific protease USP classified as a DUB protein has also been detected in *Cardinium* genome, suggesting a convergent implication of DUB in CI induced by insect endosymbionts [47]. However, some *Wolbachia* strains able to induce CI do not carry DUB domain (*i*.*e*. no *cid*) in their genomes, but display instead a paralog gene with a nuclease domain called *cinB* [34,35,38]. DUB (Cid) and Nuclease (Cin) domains do not have the same predicted functions suggesting that distinct molecular pathways may be responsible for CI [34,38]. The CI cellular defects caused by *Wolbachia* strains harboring only *cin* genes remain unknown and could differ from the one induced by *cid* genes. Our study showed that *w*Pip strains, which carry both *cid* and *cin* genes in their genomes, induce similar defects during embryogenesis as *w*Mel, which carries only a *cid* gene. This suggests that the association of *cid* and *cin* does not change the cellular phenotype of CI, but the molecular mechanism induces by DUB and Nuclease which must be different due to the biochemical nature of the proteins might converge on a similar cellular defect (*i*.*e*. paternal chromatin condensation defect). However, the presence of DUB and Nuclease domains in the same *Wolbachia* genome could still contribute to CI by modifying its penetrance: *w*Ri (*D*. *simulans*) and *w*Pip (*C*. *pipiens*), which harbor both paralogs, have indeed a strong CI penetrance (almost no hatched embryos), while *w*No and *w*Ha (*D*. *simulans*), which carry either *cin* or *cid* genes, respectively, induce lower CI penetrance [13,38].

**Fig 6:**
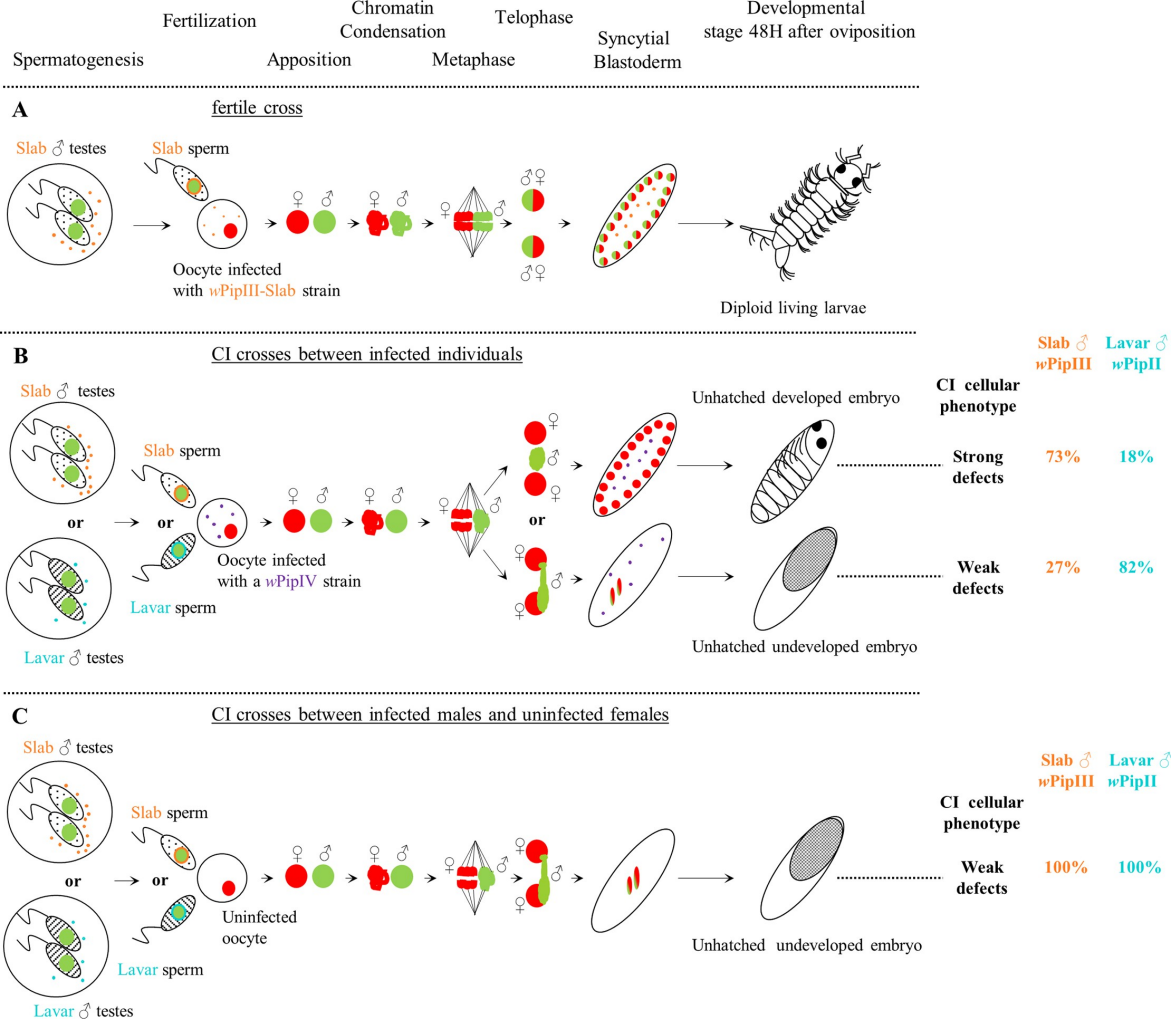
Cellular bases of different developmental fates in *C*. *pipiens*: From spermatogenesis to late development stages. Paternal and maternal DNA are represented in green and red respectively and *Wolbachia* cells are represented by the small dots (blue for *w*PipII; orange for *w*PipIII and purple for *w*PipIV). The two Mal lines Slab and Lavar have been chosen to illustrate the variability in CI defects intensity (*i*.*e*. frequency of strong and weak cellular CI). During spermatogenesis, the *Wolbachia* in male testes produce a toxin (*mod* factor). After fertilization, if females and males are infected with the same *Wolbachia* (Panel A), they can prevent the toxicity (*resc* function) and allow normal embryogenesis and the production of living diploid larvae. However, if the *Wolbachia* in the females are incompatible with the *Wolbachia* in the males (Panel B) or absent (Panel C) then the toxicity would not be prevented and paternal chromatin condensation delay and segregation defects occur. Two outcomes of the first zygotic division are possible regarding the degree of paternal chromatin exclusion. If the paternal chromatin is partially excluded, chromatin bridges would be formed resulting in aneuploid nuclei which might go through few mitotic divisions but will arrest the embryogenesis at early stages producing embryos with no visible development. If the paternal chromatin is fully excluded from the first zygotic division, maternal chromatin can segregate and produce two haploid nuclei which will divide further to produce non-viable haploid embryos exhibiting advanced development stages with eyes and segmentation clearly visible. The proportion of unhatched haploid embryos is influenced by different *w*Pip with distinct *mod* profiles in the Mal lines involved in the crosses. However when the egg is not infected by *Wolbachia* (Panel C), all embryos exhibited an absence of further development after the first zygotic divisions whatever the *mod* induced by the *w*Pip hosted by the Mal lines.

Our cytological investigation in *C*. *pipiens* evidenced a link between the paternal chromatin exclusion degree during the first zygotic division and the existence of two developmental fates following first-division defects. In fact, unhatched embryos can either reach advanced developmental stages, exhibiting segments and visible eyes, or display no visible development (Figs 2G1, 2G2 and 6) [26,44]. We confirmed Duron and Weill (2006)[44] findings that the unhatched developed embryos resulting from CI were haploid, and carried genetic material from maternal origin only (Fig 6 and S1 Fig). Confocal observations showed that such haploid development likely occurred when paternal chromatin was fully excluded during the first zygotic division, allowing the successful segregation of the isolated maternal chromatin (Fig 6). In contrast, unhatched non-developed embryos would be due to partial exclusion of the paternal chromatin, which would result in aneuploid nuclei and early arrest of embryogenesis (Fig 6). It has been previously proposed for other arthropod models that the participation of paternal chromatin to the first division would depend on the intensity of paternal chromatin defects (*i*.*e*. improper condensation) [29,30,44,48]. Severe defects would lead to complete paternal exclusion (*i*.*e*. strong cellular CI) and to the production of haploid developed embryos, while less severe defects would lead to a partial paternal chromatin exclusion (*i*.*e*. weak cellular CI) and to the production of aneuploid non-developed embryos. We used this link between the degree of paternal chromatin exclusion and the ratio of unhatched developed and non-developed embryos in eggs-rafts from incompatible crosses to investigate the variability of cellular CI intensity between different incompatible crosses.

We first studied the variability of CI intensity using males and females both infected with incompatible *w*Pip strains. Developed embryos were observed in all these incompatible crosses, with two possible outcomes: i) less than one per thousand of these embryos were apparently not affected by CI and hatched into diploid larvae [14,44], and ii) from 11% to 85% of the unhatched embryos, depending on the crosses, reached late embryonic developmental stages showing that they experienced strong CI defects (Figs 4 and 6). We then studied the influence of the absence of *Wolbachia* in the oocytes on the cellular CI intensity. As in Duron and Weill (2006)[44], i) we confirmed that not a single larvae was produced in such crosses, and ii) all the seven CI crosses between infected males and uninfected females (TC lines) resulted in 100% of non-developed embryos suggesting that in such crosses, CI phenotype was always weak (Fig 6).

In crosses between infected individuals, it clearly appeared that Mal lines harboring *Wolbachia* from different *w*Pip groups (*w*Pip I, II, III) and displaying distinct *mod* induced significant variation in CI defects intensity when crossed with females harboring distinct *w*PipIV strains displaying the same *resc* (Figs 4 and 6). Variation in CI defects intensity has already been reported in *Nasonia* species, where the production of haploid viable males in *N*. *vitripenis* was interpreted as resulting from severe paternal chromatin defects, while the production of unviable aneuploid embryos in *N*. *longicornis* and *N*. *giraulti* was interpreted as resulting from weak paternal chromatin defects. However, variation of CI intensity in these host species was not associated with the different *Wolbachia* strains, but to variation in host genetic backgrounds [19]. The backcross experiment performed in the present study suggests that CI intensity is not impacted by nuclear genetic variations in *C*. *pipiens*. While it was already established that *Wolbachia* drives alone the observed variation in crossing types in *C*. *pipiens* [11,49,50], *Wolbachia* also seems to dictate the intensity of CI defects. Consequently, the variation in CI intensity observed when two infected individuals are crossed seems to be under the major influence of the *w*Pip strain infecting the Mal line *via* the degree of paternal chromatin exclusion they trigger.

In *C*. *pipiens*, when females from tetracycline-cleared lines (TC females) were crossed with the four Mal lines, 100% of unhatched non-developed embryos only exhibiting few degenerated nuclei were observed, even 2 hours after oviposition (Figs 3D and 6). Such CI phenotype suggests that the defects caused by the *w*Pip infecting all the Mal lines are always weak (Fig 6). This result is counter intuitive because one would expect that when *Wolbachia* is absent from the eggs CI should be always strong and many haploid embryos should be produced. We mentioned above that all the Mal lines can induce strong CI defects in variable proportion of the embryos when crossed with infected females. Consequently, the constant weak CI phenotype observed when females are not infected is linked to the absence of *Wolbachia* during egg maturation. Our results suggest that in incompatible crosses between infected *C*. *pipiens* individuals, the presence of maternal *Wolbachia* somehow interferes with early embryogenesis allowing haploid development to occur. It seems very unlikely that the presence of incompatible *Wolbachia* in the egg would enhance the mechanisms leading ultimately to paternal chromosome condensation defects (*i*.*e*. accentuate the *mod* function) to result in its total exclusion during the first embryonic division. Instead, the presence of incompatible *Wolbachia* in the eggs may have an additive effect on the incompatibility between pronuclei, not by directly affecting the paternal chromatin but by influencing the cell cycle timing. For instance, maternal *Wolbachia* could modulate the maternal kinetics for DNA replication or the mitotic entry during early development, increasing the incompatibility between pronuclei and therefore favoring the haploid development. Thus, while paternal *Wolbachia-*induced CI defects always occur regardless of the infection status of the eggs, the absence of incompatible maternal *Wolbachia* would block haploid development resulting in weak CI phenotype.

We then investigated the putative genetic determinism of CI intensity variation in embryos derived from infected parents. We assessed whether it could result from difference in *Wolbachia* density, *cidA-cidB* gene expression, copy numbers, or variant diversity between the *w*Pip strains. As previously described in *Drosophila* [35,38], we found in *C*. *pipiens* that *cidA* was always significantly more expressed than *cidB*, whatever the *w*Pip strain (Table 1). This is in accordance with the hypothesis that *cidA* and *cidB* form a toxin-antidote system where CidA is the antidote of CidB [34,36]. Indeed, in such system the antidote was always found more expressed than the toxin to prevent the host from toxicity [51]. No significant difference between Mal lines was found for *cidA* and *cidB* expression levels per *Wolbachia* cell (Table 1), suggesting that the *cidA* and *cidB* expression does not influence CI defects intensity. However, while the *cidA* and *cidB* expression levels per *Wolbachia* cell did not significantly vary between *C*. *pipiens* lines, the total amount of CidA and CidB proteins in the host mainly depends on the density of *Wolbachia*. Since the *mod* factors are most likely deposited on the sperm in the testes during spermatogenesis [32,33], we measured the density of *Wolbachia* in the male gonads. We found that Lavar males hosted significantly less *Wolbachia* in their testes than males from the three other lines (Fig 5); Lavar males were also those that generated the lowest proportion of unhatched developed embryos in their offspring, whatever the Fem lines (Fig 4). Due to lower *Wolbachia* density in the testes, the global amount of CidB protein could be lower in Lavar line compared to the other lines. This low dosage of CidB would more likely result in weak CI defects leading to only few haploid development. However, this hypothesis relies on a single line and requires more *C*. *pipiens* lines with distinct testicular *Wolbachia* densities to be confirmed. Lavar was also the line with the highest *cidA* expression relatively to *cidB* (Table 1); as CidA has been proposed as the CidB antidote [34,36], its overexpression could reduce CidB-induced CI defects, and contribute to the low frequency of developed haploid embryos observed in crosses involving males from Lavar line.

We previously demonstrated that the amplification followed by the diversification of *cidA* and *cidB* variants in *w*Pip certainly constitutes the source for CI diversity profiles in *C*. *pipiens* while *cinA* and *cinB* did not exhibit any polymorphism [36]. Indeed, specific variations in *cidA* and *cidB* repertoires (number and/or nature of the variants) clearly seemed to determine the compatibility outcome of crossings between *w*PipIV-infected males and any infected females, pointing out the putative role of these variations in the prodigious CI complexity recorded in this species [36,42]. Here, we tested the putative consequence of *cidA* and *cidB* gene amplification (*i*.*e*. number of copies per genome) on variation of CI defects intensity, and demonstrated no significant correlation between the two parameters. When the quantification of genomic copies obtained by q-PCR are put in relation to the number of different variants in the same isofemale line obtained by cloning-sequencing, some of *cidA* results appear discordant. This is especially true for the Slab line, which exhibits ten distinct *cidA* variants for ~5 copies per genomes quantified (Table 1 and S2 Fig). Even taking into account technical limits of q-PCR to quantify high level of gene amplification, this discordance suggests that, at least in the Slab line, some of the *Wolbachia* cells do not harbor the same *cidA* variants.

We found that the different *w*Pip strains carried by the four Mal lines exhibiting different *mod* profiles harbored distinct *cidB* variants. Any variant of this gene could certainly trigger CI alone, as the DUB domain is perfectly conserved between all variants [36]. However, their diversity can modulate CI defects intensity. We thus tested whether *cidB* repertoire diversity could play a role in CI intensity variability. Supporting this hypothesis, we found that males from the two *C*. *pipiens* lines harboring *w*Pip strains with four different *cidB* variants induced higher proportions of unhatched developed embryos compared to lines harboring *w*Pip with only two different *cidB* variants (S4 Fig). Each distinct *cidB* variants could differentially impact the paternal chromatin (*i*.*e*. like different locks), putatively leading to an additive *mod* effect: the more different *cidB* variants present in a *w*Pip strain, the more likely strong CI defects. However, more *w*Pip strains varying in their diversity of *cidB* are required to further test this hypothesis.

In conclusion, despite the diversity of crossing types observed in *C*. *pipiens*, linked to the diversity of *cidA*/*cidB* variants repertoires, a single cellular phenotype of CI, was observed in this species. In all crosses (*i*.*e*. uni-bidirectionnal), CI results in early developmental defects in the paternal chromatin condensation and segregation during the first zygotic division similar to that observed in other insects. Our study demonstrates that in CI crosses between two infected individuals, the CI intensity (*i*.*e*. frequency of strong and weak CI defects) is influenced by the male-carried *w*Pip. However, when the female is not infected, and despite the variability of the distinct *w*Pip strains carried by the males, no unhatched developed embryos (strong cellular CI) were ever found, suggesting that the weak CI phenotype observed in such crosses is instead due to the absence of *Wolbachia* in the eggs. Genetic investigation reveals that the variability of CI defects intensity may be linked to *cidB* variant diversity in *w*Pip strains. While the putative functional role and the singularity of *cidB* amplification and diversification in *w*Pip remains yet to be fully solved, it clearly appears that it deeply modifies the *w*Pip-induced CI phenotype at different scales, from crossing types [36] to its intensity at the cellular level.

## Materials and methods

### *Culex pipiens* lines

#### *C*. *pipiens* lines hosting different *Wolbachia* strains or without *Wolbachia*

Ten laboratory isofemale lines belonging to *C*. *pipiens s*.*l*., from our laboratory, were used; they differ in their geographical origins and in the *Wolbachia* strain hosted (S1 Table). To determine the *mod* and *resc* profiles of the different mosquito lines, crosses with four reference lines (4-ref cytotypes) were realized similarly to Atyame et al. (2014)[42] (S4 Table). To study the effect of the absence of *Wolbachia* on embryogenesis, tetracycline-treated *Wolbachia*-free lines (TC lines) SlabTC, IstanbulTC, Ichkeul 21TC, and Ichkeul 13TC were obtained respectively from Slab, Istanbul, Ichkeul 21 and Ichkeul 13 *w*Pip infected lines, as described in Duron et al. (2006)[11]. After TC treatment, PCR amplifications of a fragment of *wsp* gene using the primers designed in Berticat et al. (2002)[52] allowed controlling the absence of *Wolbachia* in DNA extracted from a larvae pool (Dneasy Blood & Tissue Spin-Column protocol Kit; Qiagen; Bench protocol: Animal Tissues). To prevent possible side-effects of the treatment, TC-treated lines were raised in standard laboratory conditions for at least four generations without tetracycline before the beginning of experiments.

#### *C*. *pipiens* lines with the same host genetic background but different *Wolbachia* strains

*w*PipI strain from Tunis line was introduced into Slab line nuclear genetic background through 8 backcrosses. For the first generation, 200 females from Tunis line were crossed with 100 males of the SlabTC line. Then, for each of the seven following generations, 200 females from the previous generation were crossed with 100 males from the SlabTC line. This led to a progressive replacement (over 97%) of the maternal nuclear genome (Tunis) by the paternal nuclear genome (SlabTC), with retention of the maternal cytoplasm, including the Tunis *Wolbachia* strain [this line was called Sl(*w*PipI-Tunis)].

### Cellular study of early embryogenesis

To characterize CI cellular phenotype(s) in *C*. *pipiens*, several crosses were performed (S2 Table). For every crosses, to avoid confounding age effects, two-day old adults were released in cages. Cages containing 100 females and 50 males were then put into a closet at 25°C where day-night cycle was inverted to allow collection of early developmental stage eggs during the day. After six days in these cages, females were fed with turkey blood in heparin sodium (bcl Wholly Wild World) using a Hemotek membrane feeding system (Discovery Workshops, United Kingdom). Five days after blood meal, water-pots were placed into the cages to collect the eggs-rafts. For *C*. *pipiens* eggs, at 25°C, the meiosis is approximatively completed 30 minutes after the oviposition and the first mitotic nucleus division 15 minutes after the end of the meiosis, while four hours after oviposition the embryos normally reach the syncytial blastoderm stage [53]. Since, the CI defects described in *D*. *simulans* [25] and *N*. *vitripenis* [23,24] occurred during the first nucleus mitotic division, we mainly collected eggs aged from 30 minutes to 1hour. Older eggs were also harvested to monitor further developmental stages in both fertile and sterile crosses. Eggs-rafts were then placed into commercial bleach (active ingredient, 9.6% of sodium hypochlorite) to dissociate eggs, and then washed in distilled water. They were then fixed by being shaken for 2 hours in a solution of 3.2% para-formaldehyde in PBS 1X with Tween 0,02% (PBS-T) and washed with PBS 1X. For each fixed egg, the chorion was removed manually with a needle under an optical microscope (Leica MZ 8). Dechorionated embryos were then collected and treated with RNAse A (10 mg/mL, Sigma) overnight.

To differentially visualize the paternal from the maternal chromatin, we used propidium iodide to mark both chromatin and an anti-acetylated histone H4 labelling that preferentially marks the *de novo* assembled paternal chromatin after protamine removal [22]. Thus maternal and paternal chromatin will be respectively predominantly marked with propidium iodide (mosty red fluorescence) and with anti-acetylated histone H4 antibodies (mostly green fluorescence). For immunolabeling, embryos were first incubated overnight at 4°C with primary antibodies (Polyclonal anti-acetylated histone H4 primary antibody (1:1000, Upstate)), washed during one day with PBS-T 1X, then incubated overnight at 4°C with the secondary antibody (Alexa Fluor 488 goat anti-rabbit IgG secondary antibodies (1:250, Invitrogen)) then washed with PBS-T 1X. Embryos were then incubated in PBS-T 1X for 20 minutes with propidium iodide a DNA intercalating agent (Molecular Probes, 10μL/1mL). Finally, embryos were washed for 5 minutes and mounted between slide and coverslip in Fluoroshield Mounting Medium (Vector). Confocal microscope images were captured on an inverted photoscope (DMIRB; Leitz) equipped with a laser confocal imaging system (TCS SP5; Leica) using an HCX PL APO 1.4 NA 63 oil objective (Leica). Images from fixed, immunostained embryos are merged confocal z-stacks taken sequentially in the green and red channels for the anti-acetylated histone H4 labelling and the propidium iodide signal respectively. Crosses from which confocal microscope images were obtained (Figs 1–3) are listed in S3 Table.

### Proportion of unhatched developed embryos in CI crosses

To study the proportion of unhatched developed embryo in CI crosses, we performed a total of 32 crosses: 20 crosses involving four lines for the males (Mal lines) and five lines for the females (Fem lines), 5 involving Sl(*w*PipI-Tunis) for the males and the five Fem lines, and 7 involving the four Mal lines and females from different TC lines (S2 Table). All these crosses were performed using 50 females and 25 males. After 6 days in the cages, females were blood-fed and after 5 days eggs-rafts were collected in water pot and deposited into 24 wells plates. As hatching normally occurs approximately 48 hours after oviposition, developmental status in non-viable rafts was characterized at least two days after eggs-rafts collection. To attribute a developmental status to each egg, eggs-rafts were mounted between slide and coverslip, observed and documented with an optic microscope (Axiophot2 equipped with a CCD camera, Zeiss). Two developmental statuses were discriminated i) unhatched embryos harboring no visible development (Fig 2G1), or ii) unhatched embryos with visible development (Fig 2G2). For each cross, we calculated the proportion of embryos showing development for 50 embryos per eggs-raft in 10 eggs-rafts (total of 500 eggs observed per cross).

### Ploidy determination in CI developed embryos

To assess the ploidy status in unhatched developed embryos, we used a PCR/RFLP diagnosis *kdr/RsaI* that allowed discriminating between *C*. *pipiens* and *C*. *quinquefasciatus* lines, as previously described in Duron and Weill (2006)[44]. Slab (*C*. *quinquefasciatus)* and Ichkeul 13 (*C*. *pipiens*) were chosen because they exhibit an unidirectional sterile cross: fertile in the direction (♂Ichkeul 13 x ♀Slab) and sterile in the other direction (♂Slab x ♀Ichkeul 13). This PCR/RFLP test was performed on DNA extracted as describe above from i) a pool of larvae from Slab and Ichkeul 13 parental lines and ii) from eggs-rafts resulting from the two reciprocal crosses between those two lines.

### Real Time Quantitative PCR

#### Quantification of *Wolbachia* density in male testes

In order to test the influence of *Wolbachia* densities in testes on the CI cellular intensity, we quantified them with Real Time Quantitative PCR using the LightCycler 480 system (Roche). Specific primers and procedures were described in Berticat et al. (2002)[52]. Testes from 6-day old males of Tunis, Utique, Lavar and Slab lines were sampled. Each DNA template were obtained from pools of three testis pairs and extracted as described above. Five independent DNA templates were realized for each line. To estimate the number of *Wolbachia* per mosquito testes, we amplified two different genes on each sample, the *C*. *pipiens* specific *ace-2* locus [54] and the *Wolbachia* specific monocopy *wsp* locus [52]. Standard curves were performed using dilutions of a pBluescriptKS vector containing a unique *ace-2* and *wsp* gene copy. Each DNA template was analyzed in triplicate for both *wsp* and *ace-2* quantification. As both genes are present as single copies per haploid genome, the ratio of *wsp* over *ace-2* signals allowed estimating the relative number of *Wolbachia* genomes per *Culex* genome, thus correcting for mosquito size and DNA quality.

#### Amplification of *cidA* and *cidB* genes within *w*Pip genome

For each *C*. *pipiens*-*Wolbachia* line (Tunis, Utique, Lavar and Slab hosting a different *w*Pip strains belonging to group I, II or III) and exhibiting different *mod* profiles, quantitative PCRs were carried out to estimate the number of copies of *cidA* and *cidB* genes per *w*Pip genome. Three different quantitative PCRs were performed on DNA samples extracted from ten 6-day old males per line following the procedure described in Berticat et al. (2002)[52]: i) specific of the locus *wsp*, ii) specific of a 189bp fragment of the *cidA* gene conserved between all *w*Pip strains using primers *w*Pip_0282_QPCR_2_Dir (5’-AGG-TCC-TGT-ATT-TGA-TTT-CTG-GA) and *w*Pip_0282_QPCR_2_Rev (5’-TGA-ACG-CGA-GAA-AGA-GCA-AG), and iii) specific of a 135bp fragment of the *cidB* gene conserved between all *w*Pip strains using primers *w*Pip_0283_QPCR_1_Dir (5’-TGA-GTG-TTT-GGA-GAA-TGA-AGG-A) and *w*Pip_0283_QPCR_1_Rev (5’-TTC-CCA-AAA-GCA-AAA-CCA-GTT). Standard curves of *cidA*, *cidB* and *wsp* genes were performed using dilution of the PCR product of these three genes previously quantified using the flurorometre-QuBit (Invitrogen). Each DNA template was analysed in triplicate for *wsp*, *cidA* and *cidB* locus. *CidA* and *cidB* copy numbers were estimated using the ratio of *cidA* or *cidB* estimated copy number over *wsp* estimated copy number, to obtain a copy number per *Wolbachia* genome since *wsp* is present in one copy in *Wolbachia* genome.

#### Expression of *cidA* and *cidB* genes

For each line Tunis, Utique, Lavar and Slab, ten 6-day old males were used for RNA extraction with Trizol (Life Technologies) and treated with DNase with the TURBO DNA-free Kit (Life Technologies), in accordance with the manufacturer's instructions. 2–5 μg of each total RNA sample were reverse-transcribed into cDNA with the SuperScript III Reverse Transcriptase Kit and 30 ng of random oligomer primers ((RP)10; Invitrogen, Life Technologies). Three different quantitative PCRs were performed: i) specific of *wsp* locus, ii) specific of *cidA* locus, and iii) specific of *cidB* locus as describe above. Each DNA template was analysed in triplicate for *wsp cidA* and *cidB* locus. Levels of expression of *cidA* and *cidB* genes were estimated relatively to *wsp* genes by using the ratio of expression of these two genes over *wsp*.

### Determination of *cidA*/*cidB* variants repertoire in the different *w*Pip hosted by males

To describe the diversity of *cidA*^*w*Pip^/*cidB*^*w*Pip^ repertoires for the two *C*. *pipiens* lines Utique and Slab not yet investigated, cloning and Sanger sequencing of the *cidA* and *cidB* variants were performed as described in Bonneau et al. (2018)[36] on DNA from pools of larvae extracted as described above. Variant sequences were aligned, using the Muscle algorithm implemented in Seaview 6.4.1 software [55].

### Statistical analysis

Variability of unhatched developed embryo proportion in sterile crosses was analyzed using a generalized linear model (GLM): Udep = Cross + ε, with Udep the proportion of unhatched developed embryos for each cross (Cross, which represent the interaction between the Mal and Fem lines) and ε the error parameter, following a binomial distribution. To test the specific effect of the four Mal lines and the five Fem lines separately, GLMs with mixed effects (GLMM) were used: Udep = Male + Female + 1|Cross + ε with Male and Female respectively the Mal and Fem lines involved in each cross as fixed effects, with Cross as the interaction between Mal and Fem lines as a random effect (as crosses to produce embryos necessary require an interaction between females and males), and ε the error parameter, following a binomial distribution. To test for a specific effect of the host genetic background in crosses involving males from Sl(*w*PipI-Tunis) and Tunis lines which host the same *Wolbachia* in two different genetic background we used a GLMM: Udep = MalBack + Female + 1|Cross + ε with Udep the unhatched developed embryos proportion for each cross involving males from Sl(*w*PipI-Tunis) and Tunis lines (MalBack) and the five Fem lines (Female) as fixed effects, with Cross as a random effect, and with ε the error parameter, following a binomial distribution.

For several variables (*Wolbachia* density in testes, *cidA* and *cidB* expressions and copy number) obtained with q-PCR, variability between the four Mal lines was analyzed using GLMs in the form Var = Male + ε, with Var one of the estimated variable of the Mal line (Male) and ε the error parameter, following a Gaussian distribution.

Spearman correlation tests [56] were used to test for correlation between these variables (*Wolbachia* density in testes, *cidA* and *cidB* expressions and copy number) and the proportion of unhatched developed embryos for each Mal line. We did the same for the relation between the number of different *cidA* variants and the proportion of unhatched developed embryos for each Mal line. Finally, Wilcoxon test [57] was used to compare mean proportions of unhatched developed embryos between the two Mal lines harboring only two different *cidB* variants and the two Mal lines harboring four different *cidB* variants.

All computations were performed using the R version 3.4.4 [58]. Computed models were simplified by testing the significance of the different terms using likelihood ratio tests (LRT) and starting from the higher-order terms, as described in Crawley [59]. Factor levels of qualitative variables that were not different in their estimates (using LRTs) were grouped as described by Crawley [59]. The normality of the residuals was tested using Shapiro test for models with Gaussian error [60]. For models with Binomial error, overdispersion was calculated using the “dispersion_glmer” function from the package blmeco for GLMM, and by dividing the residual deviance by the residuals degree of freedom of the model for GLM [61]; when detected, overdispersion was taken into account in the LRTs [62,63].

## Supporting information

S1 Table Presentation of the ten *C*. *pipiens* lines used in this study. (DOCX)

S2 Table The different crosses from which the CI cellular phenotype in *C*. *pipiens* was studied. Three different types of crosses were performed to study the cellular phenotype responsible for embryonic death in sterile crosses: i) sterile crosses between males and females infected with different *Wolbachia* strains, ii) sterile crosses between infected males and uninfected females, and iii) fertile crosses between males and females from the same mosquito line infected or not by *Wolbachia*. Crosses from which confocal and optical microscopy pictures were taken are indicated next to the cross (Figs 1–3).
(DOCX)

S3 Table Proportion of unhatched developed embryos from CI crosses involving either infected or uninfected (TC) females. Proportion of unhatched developed embryos are given as the mean proportion measured on fifty eggs for 10 rafts per cross (500 eggs observed per cross) ± standard deviation. No unhatched developed embryos were found in any of the seven different crosses performed between infected males and uninfected females.
(DOCX)

S4 Table *mod* and *resc* profiles of the lines used in crosses experiments. *mod* profiles were determined by crossing males from the four Mal lines with the females of the 4 ref-cytotypes lines Atyame et al. (2014). *resc* profiles were determine by crossing females of the five Fem lines with males of the 4 ref-cytotypes lines. Mal lines harbored *w*Pip strains with different *mod* profiles while *w*Pip strains from the five Fem lines presented the same *resc* profile.
(DOCX)

S5 Table Accession numbers. Accession numbers for *cidA cidB* variants analyzed in S2 and S3 Figs.
(DOCX)

S6 Table Explanation of the nomenclature used in this paper. (DOCX)

S1 Fig Unhatched developed embryos are haploid. Restriction profile of *kdr* PCR products by *RsaI* enzyme from single mosquito extracted DNA. M: molecular weight marker. **1** *w*PipIII-Slab line; **2/3**: *w*PipIV-Ichkeul 13 line; **4/5**: eggs-raft containing non-viable developed embryos from a CI cross between ♂ Slab x ♀ Ichkeul 13 (embryos display only maternal markers); **6**: eggs-raft containing viable embryos from the fertile cross between ♂ Ichkeul 13 x ♀ Slab.
(TIF)

S2 Fig Repertoires of CidA protein variants in the four Mal line *w*Pip strains. Protein sequences alignment of the CidA variants found in the four *Wolbachia* strains *w*PipI-Tunis, *w*PipI-Utique, *w*PipII-Lavar and *w*PipIII-Slab (Mal lines). The first sequence is used as a reference to determine the polymorphic region. For more clarity, only polymorphic positions are represented, thus amino-acid positions are not continuous. When more than two contiguous amino-acids were variable the “-”symbol was used between the first and the last variable position of the zone. Colors show polymorphic blocks of residues present in variants regardless of their phylogenetic *w*Pip group (I to III). No *cidA* or *cidB* nucleotide sequence variant was shared between the three *w*Pip groups. However, the *w*PipII-Lavar CidA_II(α/1) variant and the *w*PipIII-Slab CidA_III(β/8) variant presented the same amino-acid sequence. Based on their nucleotide sequences *w*PipIII-Slab exhibited ten variants of *cidA*, *w*PipII-Lavar three, *w*PipI-Tunis four and *w*PipI Utique seven. However, *w*PipIII-Slab exhibited only seven variants that differ in their amino-acid sequences since *cidA*_III(χ/6) and *cidA*_III(χ/7), *cidA*_III(δ/6) and *cidA*_III(δ/7), *cidA*_III(δ/8) and *cidA*_III(δ/9) have respectively identical amino-acid sequences (*i*.*e*. nucleotide polymorphic positions between them are synonymous).
(TIF)

S3 Fig The repertoire of CidB protein variants in the four Mal line *w*Pip strains. Protein sequences alignment of the CidB variants found in the four *Wolbachia* strains *w*PipI-Tunis, *w*PipI-Utique, *w*PipII-Lavar and *w*PipIII-Slab (Mal lines). The first sequence is used as a reference to determine the polymorphic region. For more clarity, only polymorphic positions are represented, thus amino-acid positions are not continuous. When more than two contiguous amino-acids were variable the “-”symbol was used between the first and the last variable position of the zone. Colors show polymorphic blocks of residues present in variants regardless of their phylogenetic *w*Pip group (I to III). However, no variant (*i*.*e*. complete CidB sequence) is common to *w*Pip strains from different groups.
(TIF)

S4 Fig Correlation between *cidB* variants diversity in *w*Pip genomes and CI cellular intensity. Lighter gray bar plot accounts for the 10 crosses involving males from Lavar and Utique lines hosting *w*Pip, which harbor 2 different variants of *cidB* in their genomes, while darker gray bar plot accounts for the 10 crosses involving males from Tunis and Slab lines, both infected with *w*Pip strains harboring 4 different variants of *cidB*. Error bars represent the standard error. The proportion of unhatched developed embryos was significantly higher for males hosting four-variants *w*Pip strains than for males hosting two-variants *w*Pip strains (Wilcoxon, W = 1159, p<0.001).
(TIF)

S5 Fig *cidA* copy number in the *w*Pip strains infecting the four Mal lines. *cidA* copy number was measured by quantitative PCR as the ratio between the number of copies of the *Wolbachia cidA* gene and the *Wolbachia wsp* gene. The colored dots represent the *cidA* copy number per *w*Pip genome in a male and the red strips represent the average *cidA* copy number per *w*Pip genome for ten males per Mal lines. Letters represent the different statistical groups (*i*.*e*. means with the same letter are not significantly different).
(TIF)

S6 Fig *cidB* copy number in the *w*Pip strains infecting the four Mal lines. *cidB* copy number was measured by quantitative PCR as the ratio between the number of copies of the *Wolbachia cidB* gene and the *Wolbachia wsp* gene. The colored dots represent the *cidB* copy number per *w*Pip genome in a male and the red strips represent the average *cidB* copy number per *w*Pip genome for ten males per Mal lines. Letters represent the different statistical groups (*i*.*e*. means with the same letter are not significantly different).
(TIF)

S7 Fig *cidA*/*cidB* copy number in the *w*Pip strains infecting the four Mal lines. *cidA*/*cidB* copy number was measured by quantitative PCR as the ratio between the number of copies of the *Wolbachia cidA* gene and the *Wolbachia cidB* gene. The colored dots represent the *cidA*/*cidB* copy number per *w*Pip genome in a male and the red strips represent the average *cidA*/*cidB* copy number per *w*Pip genome for ten males per Mal lines. *cidA*/*cidB* copy number were not significantly different between the four *w*Pip strains infecting the four Mal lines.
(TIFF)

S8 Fig *cidA* expression level in the *w*Pip strains infecting the four Mal lines. *cidA* expression was measured by quantitative PCR as the ratio between the *Wolbachia cidA* gene expression and the *Wolbachia wsp* gene expression. The colored dots represent the *cidA* expression level per *w*Pip genome in a male and the red strips represent the average *cidA* expression level per *w*Pip genome for ten males per Mal lines. Expression levels of *cidA* genes were not significantly different between the four *w*Pip strains infecting the four Mal lines.
(TIFF)

S9 Fig *cidB* expression level in the *w*Pip strains infecting the four Mal lines. *cidB* expression was measured by quantitative PCR as the ratio between the *Wolbachia cidB* gene expression and the *Wolbachia wsp* gene expression. The colored dots represent the *cidB* expression level per *w*Pip genome in a male and the red strips represent the average *cidB* expression level per *w*Pip genome for ten males per Mal lines. Expression levels of *cidB* genes were not significantly different between the four *w*Pip strains infecting the four Mal lines.
(TIFF)

S10 Fig *cidA*/*cidB* expression level in the *w*Pip strains infecting the four Mal lines. *cidA*/*cidB* expression levels was measured by quantitative PCR as the ratio between the number of copies of the *Wolbachia cidA* gene and the *Wolbachia cidB* gene. The colored dots represent the *cidA*/*cidB* expression level per *w*Pip genome in a male and the red strips represent the average *cidA*/*cidB* expression per *w*Pip genome for ten males per Mal lines. Letters represent the different statistical groups (*i*.*e*. means with the same letter are not significantly different).
(TIFF)

S1 Dataset Proportion of unhatched developed haploid embryos of performed CI crosses. (CSV)

S2 Dataset *Wolbachia* testes density of the four Mal lines estimated with Real Time Quantitative PCR. (CSV)

S3 Dataset *Wolbachia cidA* and *cidB* copy number of the four Mal lines estimated with Real Time Quantitative PCR. (CSV)

S4 Dataset *Wolbachia cidA* and *cidB* expression of the four Mal lines estimated with Real Time Quantitative PCR. (CSV)
