# Supplementary material for: The cellular phenotype of cytoplasmic incompatibility in Culex pipiens in the light of cidB diversity
Source: PLoS Pathog. 2018 Oct 15;14(10):e1007364. doi: 10.1371/journal.ppat.1007364 (PMC6201942; doi:10.1371/journal.ppat.1007364)
Supplement: S1 Table — (DOCX) [file ppat.1007364.s001.docx]

| Line | *Wolbachia* group | *Culex* subspecies | Country | Year of collection | Reference |
| --- | --- | --- | --- | --- | --- |
| Tunis | *w*PipI | *pipiens* | Tunisia | 1995 | Ben Cheikh *et al*., 1998 |
| Utique | *w*PipI | *pipiens* | Tunisia | 2014 | This study |
| Lavar | *w*PipII | *pipiens* | France | 2003 | Duron *et al*., 2005 |
| Slab | *w*PipIII | *quinquefasciatus* | USA | 1954 | Georghiou *et al*., 1966 |
| Maclo | *w*PipIII | *quinquefasciatus* | USA | 1984 | Duron *et al*., 2006 |
| Istanbul | *w*PipIV | *pipiens* | Turkey | 2003 | Duron *et al*., 2005 |
| Harash | *w*PipIV | *pipiens* | Algeria | 2006 | Alout *et al*., 2009 |
| Ichkeul 09 | *w*PipIV | *pipiens* | Tunisia | 2010-2011 | Bonneau *et al*., 2018 |
| Ichkeul 13 | *w*PipIV | *pipiens* | Tunisia | 2010-2011 | Bonneau *et al*., 2018 |
| Ichkeul 21 | *w*PipIV | *pipiens* | Tunisia | 2010-2011 | Bonneau *et al*., 2018 |
