# Supplementary material for: The cellular phenotype of cytoplasmic incompatibility in Culex pipiens in the light of cidB diversity
Source: PLoS Pathog. 2018 Oct 15;14(10):e1007364. doi: 10.1371/journal.ppat.1007364 (PMC6201942; doi:10.1371/journal.ppat.1007364)
Supplement: S2 Table — Three different types of crosses were performed to study the cellular phenotype responsible for embryonic death in sterile crosses: i) sterile crosses between males and females infected with different Wolbachia strains, ii) sterile crosses between infected males and uninfected females, and iii) fertile crosses between males and females from the same mosquito line infected or not by Wolbachia. Crosses from which confocal and optical microscopy pictures were taken are indicated next to the cross (Figs 1–3). (DOCX) [file ppat.1007364.s002.docx]

| **Inter-lines CI crosses** | | **Intra-line fertile crosses** | |
| --- | --- | --- | --- |
| Between infected males and infected females | Between infected males and uninfected females | Between infected males and infected females | Between uninfected males and uninfected females |
| ♂ Utique x ♀ Harash (Fig 2F)  ♂ Utique x ♀ Istanbul (Fig 2G1 & 2G2)  ♂ Utique x ♀ Ichkeul 09 (Fig 2C)  ♂ Tunis x ♀ Ichkeul 21  ♂ Lavar x ♀ Slab (Fig 2D & 2E)  ♂ Ichkeul 21 x ♀ Utique (Fig 2A & 2B)  ♂ Ichkeul 09 x ♀ Tunis  ♂ Ichkeul 09 x ♀ Utique | ♂ Utique x ♀ IstanbulTC (Fig 3B & 3C & 3D)  ♂ Lavar x ♀ SlabTC (Fig 3A & 3E ) | ♂ Tunis x ♀ Tunis (Fig1C)  ♂ Harash x ♀ Harash (Fig 1G)  ♂ Istanbul x ♀ Istanbul (Fig 1F)  ♂ Slab x ♀ Slab (Fig 1B & 1 E)  ♂ Maclo x ♀ Maclo | ♂ SlabTC x ♀ SlabTC (Fig 1A & 1D)  ♂ Istanbul TC x ♀ Istanbul TC |
