# Supplementary material for: The cellular phenotype of cytoplasmic incompatibility in Culex pipiens in the light of cidB diversity
Source: PLoS Pathog. 2018 Oct 15;14(10):e1007364. doi: 10.1371/journal.ppat.1007364 (PMC6201942; doi:10.1371/journal.ppat.1007364)
Supplement: S3 Table — Proportion of unhatched developed embryos are given as the mean proportion measured on fifty eggs for 10 rafts per cross (500 eggs observed per cross) ± standard deviation. No unhatched developed embryos were found in any of the seven different crosses performed between infected males and uninfected females. (DOCX) [file ppat.1007364.s003.docx]

| **Crosses** | | **unhatched developed embryos rate** |
| --- | --- | --- |
| ♂ Tunis | ♀ Harash | 0.850 ± 0.153 |
| ♂ Tunis | ♀ Ichkeul 09 | 0.740 ± 0.139 |
| ♂ Tunis | ♀ Ichkeul 13 | 0.530 ± 0.179 |
| ♂ Tunis | ♀ Ichkeul 13TC | 0.000 ± 0.000 |
| ♂ Tunis | ♀ Ichkeul 21 | 0.686 ± 0.207 |
| ♂ Tunis | ♀ Ichkeul 21TC | 0.000 ± 0.000 |
| ♂ Tunis | ♀ Istanbul | 0.784 ± 0.148 |
| ♂ Tunis | ♀ IstanbulTC | 0.000 ± 0.000 |
| ♂ Tunis | ♀ SlabTC | 0.000 ± 0.000 |
| ♂ Sl(*w*PipI-Tunis) | ♀ Harash | 0.816 ± 0.056 |
| ♂ Sl(*w*PipI-Tunis) | ♀ Ichkeul 09 | 0.776 ± 0.107 |
| ♂ Sl(*w*PipI-Tunis) | ♀ Ichkeul 13 | 0.536 ± 0.338 |
| ♂ Sl(*w*PipI-Tunis) | ♀ Ichkeul 21 | 0.600 ± 0.156 |
| ♂ Sl(*w*PipI-Tunis) | ♀ Istanbul | 0.842 ± 0.122 |
| ♂ Utique | ♀ Harash | 0.440 ± 0.374 |
| ♂ Utique | ♀ Ichkeul 09 | 0.466 ± 0.265 |
| ♂ Utique | ♀ Ichkeul 13 | 0.422 ± 0.392 |
| ♂ Utique | ♀ Ichkeul 21 | 0.436 ± 0.264 |
| ♂ Utique | ♀ Istanbul | 0.330 ± 0.250 |
| ♂ Utique | ♀ SlabTC | 0.000 ± 0.000 |
| ♂ Lavar | ♀ Harash | 0.136 ± 0.093 |
| ♂ Lavar | ♀ Ichkeul 09 | 0.242 ± 0.118 |
| ♂ Lavar | ♀ Ichkeul 13 | 0.128 ± 0.138 |
| ♂ Lavar | ♀ Ichkeul 21 | 0.268 ± 0.111 |
| ♂ Lavar | ♀ Istanbul | 0.116 ± 0.082 |
| ♂ Lavar | ♀ SlabTC | 0.000 ± 0.000 |
| ♂ Slab | ♀ Harash | 0.722 ± 0.148 |
| ♂ Slab | ♀ Ichkeul 09 | 0.664 ± 0.178 |
| ♂ Slab | ♀ Ichkeul 13 | 0.662 ± 0.116 |
| ♂ Slab | ♀ Ichkeul 21 | 0.834 ± 0.069 |
| ♂ Slab | ♀ Istanbul | 0.754 ± 0.201 |
| ♂ Slab | ♀ SlabTC | 0.000 ± 0.000 |
