# Supplementary material for: The cellular phenotype of cytoplasmic incompatibility in Culex pipiens in the light of cidB diversity
Source: PLoS Pathog. 2018 Oct 15;14(10):e1007364. doi: 10.1371/journal.ppat.1007364 (PMC6201942; doi:10.1371/journal.ppat.1007364)
Supplement: S4 Table — mod profiles were determined by crossing males from the four Mal lines with the females of the 4 ref-cytotypes lines Atyame et al. (2014). resc profiles were determine by crossing females of the five Fem lines with males of the 4 ref-cytotypes lines. Mal lines harbored wPip strains with different mod profiles while wPip strains from the five Fem lines presented the same resc profile. (DOCX) [file ppat.1007364.s004.docx]

| **4-ref cytotypes** ♀  **Mal Lines** | **Lavar** | **Maclo** | **Slab** | **Istanbul** | ***mod* type** | **Reference** |
| --- | --- | --- | --- | --- | --- | --- |
| *w*PipI-Tunis | IC | C | IC | IC | ii | Atyame *et al*., 2014 |
| *w*PipI-Utique | C | IC | C | IC | iv | This study |
| *w*PipII-Lavar | C | C | IC | IC | vi | Atyame *et al*., 2014 |
| *w*PipIII-Slab | IC | C | C | IC | iii | Atyame *et al*., 2014 |
| **4-ref cytotypes** ♂  **Fem Lines** | **Lavar** | **Maclo** | **Slab** | **Istanbul** | ***resc* type** | **Reference** |
| *w*PipIV-Harash | IC | C | IC | C | 3 | Atyame *et al*., 2014 |
| *w*PipIV-Istanbul | IC | C | IC | C | 3 | Atyame *et al*., 2014 |
| *w*PipIV-Ichkeul 13 | IC | C | IC | C | 3 | This study |
| *w*PipIV-Ichkeul 09 | IC | C | IC | C | 3 | This study |
| *w*PipIV-Ichkeul 21 | IC | C | IC | C | 3 | This study |
