# Supplementary material for: The cellular phenotype of cytoplasmic incompatibility in Culex pipiens in the light of cidB diversity
Source: PLoS Pathog. 2018 Oct 15;14(10):e1007364. doi: 10.1371/journal.ppat.1007364 (PMC6201942; doi:10.1371/journal.ppat.1007364)
Supplement: S5 Table — Accession numbers for cidA cidB variants analyzed in S2 and S3 Figs. (DOCX) [file ppat.1007364.s005.docx]

| **Gene name** | **Variant sequence submission name** | **Accession number** | **Gene name** | **Variant sequence submission name** | **Accession number** |
| --- | --- | --- | --- | --- | --- |
| *cidA* | cidA_I(alpha/1) | MF444963 | *cidB* | cidB_I(a/1) | MF444982 |
|  | cidA_I(alpha/2) | MH544806 |  | cidB_I(a/2) | MF444983 |
|  | cidA_I(beta/2) | MF444966 |  | cidB_I(b/1) | MF444984 |
|  | cidA_I(gamma/1) | MF444964 |  | cidB_I(b/2) | MF444985 |
|  | cidA_I(gamma/2) | MF444965 |  | cidB_II(a/1) | MF444987 |
|  | cidA_I(zeta/1) | MH544807 |  | cidB_II(a/2) | MF444986 |
|  | cidA_I(zeta/2) | MH544808 |  | cidB_III(d/1) | MH544820 |
|  | cidA_I(zeta/5) | MH544809 |  | cidB_III(d/2) | MH544821 |
|  | cidA_II(alpha/1) | MF444967 |  | cidB_III(e/1) | MH544822 |
|  | cidA_II(alpha/2) | MF444968 |  | cidB_III(e/2) | MH544823 |
|  | cidA_II(beta/2) | MF444969 |  |  |  |
|  | cidA_III(beta/8) | MH544810 |  |  |  |
|  | cidA_III(gamma/4) | MH544811 |  |  |  |
|  | cidA_III(gamma/5) | MH544812 |  |  |  |
|  | cidA_III(gamma/6) | MH544813 |  |  |  |
|  | cidA_III(gamma/7) | MH544814 |  |  |  |
|  | cidA_III(gamma/8) | MH544815 |  |  |  |
|  | cidA_III(delta/6) | MH544816 |  |  |  |
|  | cidA_III(delta/7) | MH544817 |  |  |  |
|  | cidA_III(delta/8) | MH544818 |  |  |  |
|  | cidA_III(delta/9) | MH544819 |  |  |  |
