# Supplementary material for: The cellular phenotype of cytoplasmic incompatibility in Culex pipiens in the light of cidB diversity
Source: PLoS Pathog. 2018 Oct 15;14(10):e1007364. doi: 10.1371/journal.ppat.1007364 (PMC6201942; doi:10.1371/journal.ppat.1007364)
Supplement: S6 Table — (DOCX) [file ppat.1007364.s006.docx]

| DUB | Deubiquitylating enzyme: enzyme that remove ubiquitin from ubiquitinated protein. |
| --- | --- |
| CI penetrance | Percentage of embryos that will die from CI |
| CI cellular phenotypes | Cellular consequences of CI during early embryogenesis |
| CI intensity at the cellular level | Severity of the defect suffered by the paternal chromatin resulting in its partial or total exclusion during first embryonic division |
| *cidA* | *cidA* gene is involved in CI induction in *Drosophila melanogaster* transinfected males [35,37] and in CI rescue in *D. melanogaster* females [40]. This gene is putatively involved in CI diversity in *Culex pipiens* [36] |
| CidA | The protein produce by *cidA* gene |
| *cidB* | *cidB* gene is involved in CI induction in *D. melanogaster* transinfected males and CI diversity in *C. pipiens* [35,37,36]. |
| CidB | The protein produce by *cidB* gene |
| *cidA^w^*^Pip^, *cidA^w^*^Mel^ | *cidA* homologues respectively found in the *w*Pip and *w*Mel genomes. Protein sequences are divergent (66% of identity). |
| *cidB^w^*^Pip^, *cidB^w^*^Mel^ | *cidB* homologues respectively found in the *w*Pip and *w*Mel genomes. Protein sequences are divergent (76% of identity). |
| *cinA* | Paralogue of *cidA* gene likely involved in CI [34, 37] but not in *C. pipiens* CI diversity [36]. |
| *cinB* | Paralogue of the *cidB* gene likely involved in CI [34, 37] but not in CI diversity in *C. pipiens* [36]. |
| *Culex pipiens* crossing type | Compatibility and incompatibility relationships between males and females from different *C. pipiens* lines. |
| MLST | Multi Locus Sequence Typing: list of genes created by Baldo et al. 2006 that are used to classify the *Wolbachia* strains into the different super-groups. |
| MLST specific to *w*Pip | Group of hypervariable genes that are used to classify the different *w*Pip strains in the five *w*Pip phylogenetic groups [12]. |
| *Mod* profile | Compatibility or incompatibility profile when males from a given line are crossed with females from other lines. |
| *Resc* profile | Compatibility or incompatibility profile when females from a given line are crossed with males from other lines. |
| Toxin-antidote system | Theoretical model on CI mechanism proposed by Hurst (1991) |
| WO phage | Bacteriophage name WO after *Wolbachia* that is present as a prophage in almost all *Wolbachia* genomes. |
| *w*Mel | *Wolbachia* strains infecting *Drosophila melanogaster* |
| *w*Pip | *Wolbachia* strains infecting *Culex pipiens* |
| *w*PipI, *w*PipII, *w*PipIII, *w*PipIV and *w*PipV | The different phylogenetic *w*Pip groups obtained from *w*Pip specific MLST |
| *w*Ri, *w*No, *w*Ha | *Wolbachia* strains infecting *Drosophila simulans* |
| *w*VitA | *Wolbachia* strain infecting *Nasonia vitripenis* |
