# Supplementary figures and images for: The cellular phenotype of cytoplasmic incompatibility in Culex pipiens in the light of cidB diversity
Source: PLoS Pathog. 2018 Oct 15;14(10):e1007364. doi: 10.1371/journal.ppat.1007364 (PMC6201942; doi:10.1371/journal.ppat.1007364)

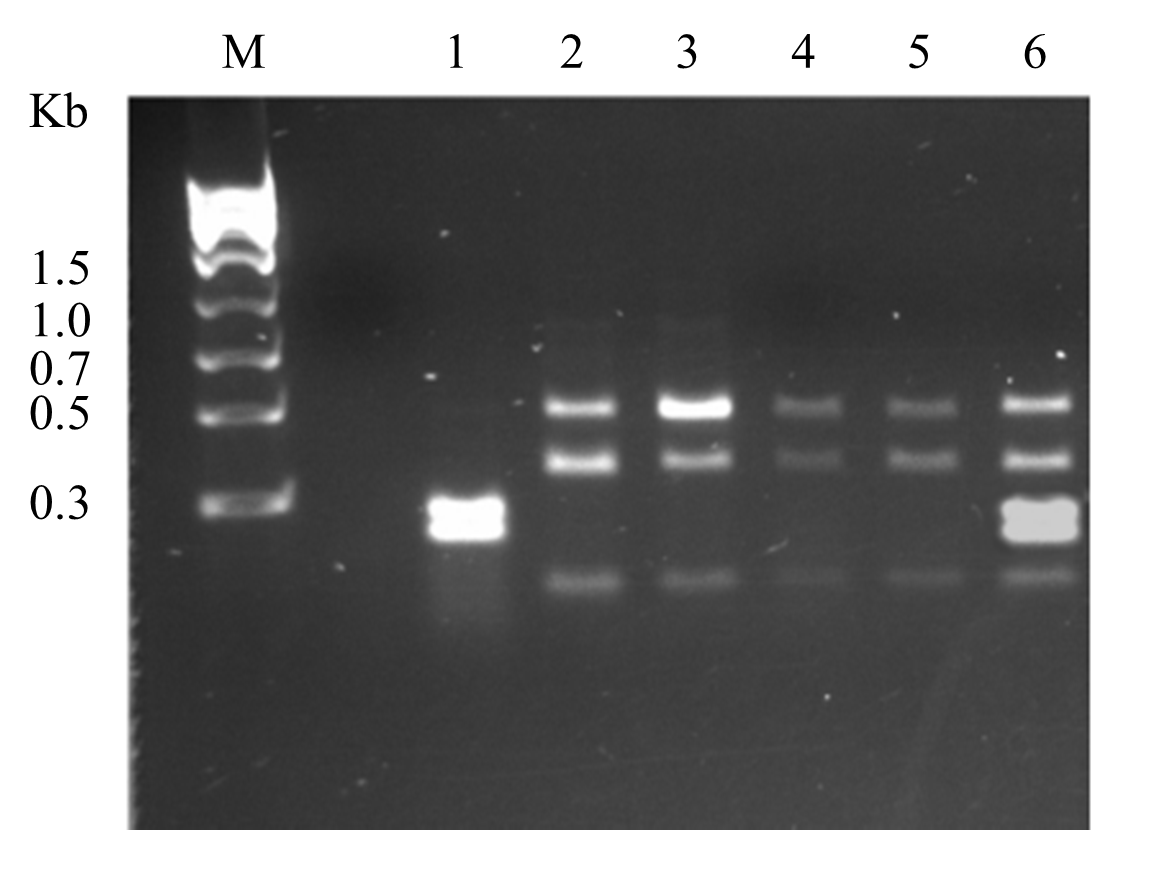

Supplement: S1 Fig — Restriction profile of kdr PCR products by RsaI enzyme from single mosquito extracted DNA. M: molecular weight marker. 1 wPipIII-Slab line; 2/3: wPipIV-Ichkeul 13 line; 4/5: eggs-raft containing non-viable developed embryos from a CI cross between ♂ Slab x ♀ Ichkeul 13 (embryos display only maternal markers); 6: eggs-raft containing viable embryos from the fertile cross between ♂ Ichkeul 13 x ♀ Slab. (TIF) [file ppat.1007364.s007.tif]

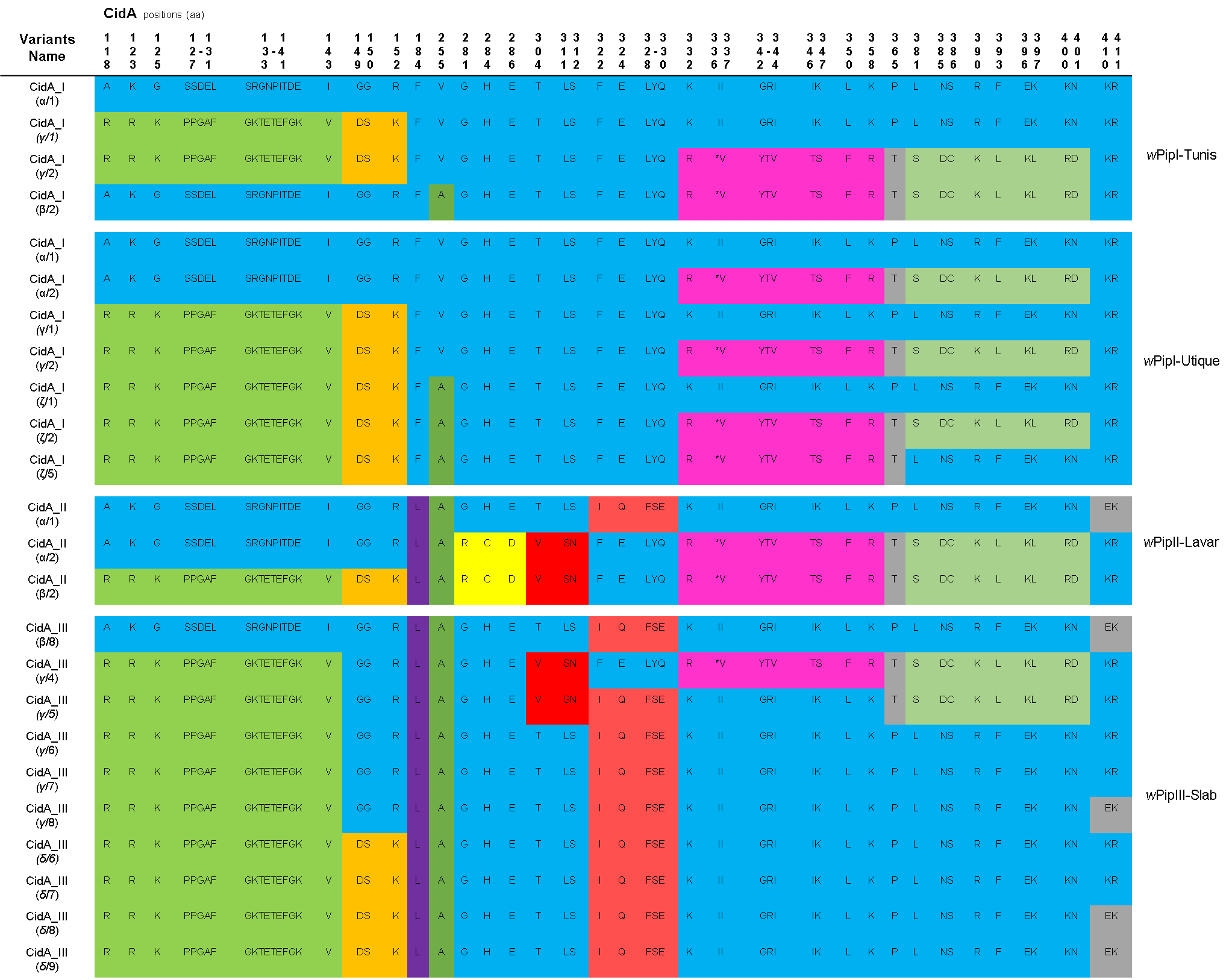

Supplement: S2 Fig — Protein sequences alignment of the CidA variants found in the four Wolbachia strains wPipI-Tunis, wPipI-Utique, wPipII-Lavar and wPipIII-Slab (Mal lines). The first sequence is used as a reference to determine the polymorphic region. For more clarity, only polymorphic positions are represented, thus amino-acid positions are not continuous. When more than two contiguous amino-acids were variable the “-”symbol was used between the first and the last variable position of the zone. Colors show polymorphic blocks of residues present in variants regardless of their phylogenetic wPip group (I to III). No cidA or cidB nucleotide sequence variant was shared between the three wPip groups. However, the wPipII-Lavar CidA_II(α/1) variant and the wPipIII-Slab CidA_III(β/8) variant presented the same amino-acid sequence. Based on their nucleotide sequences wPipIII-Slab exhibited ten variants of cidA, wPipII-Lavar three, wPipI-Tunis four and wPipI Utique seven. However, wPipIII-Slab exhibited only seven variants that differ in their amino-acid sequences since cidA_III(χ/6) and cidA_III(χ/7), cidA_III(δ/6) and cidA_III(δ/7), cidA_III(δ/8) and cidA_III(δ/9) have respectively identical amino-acid sequences (i.e. nucleotide polymorphic positions between them are synonymous). (TIF) [file ppat.1007364.s008.tif]

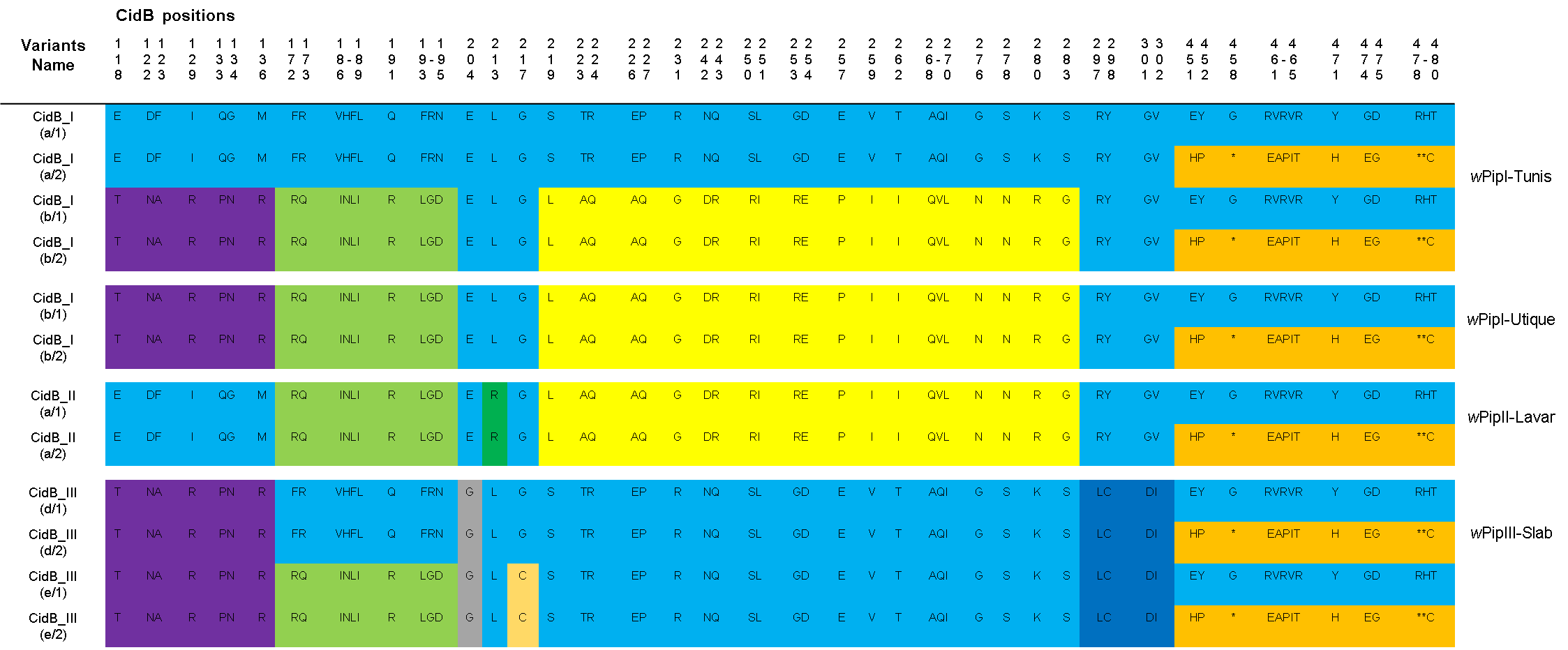

Supplement: S3 Fig — Protein sequences alignment of the CidB variants found in the four Wolbachia strains wPipI-Tunis, wPipI-Utique, wPipII-Lavar and wPipIII-Slab (Mal lines). The first sequence is used as a reference to determine the polymorphic region. For more clarity, only polymorphic positions are represented, thus amino-acid positions are not continuous. When more than two contiguous amino-acids were variable the “-”symbol was used between the first and the last variable position of the zone. Colors show polymorphic blocks of residues present in variants regardless of their phylogenetic wPip group (I to III). However, no variant (i.e. complete CidB sequence) is common to wPip strains from different groups. (TIF) [file ppat.1007364.s009.tif]

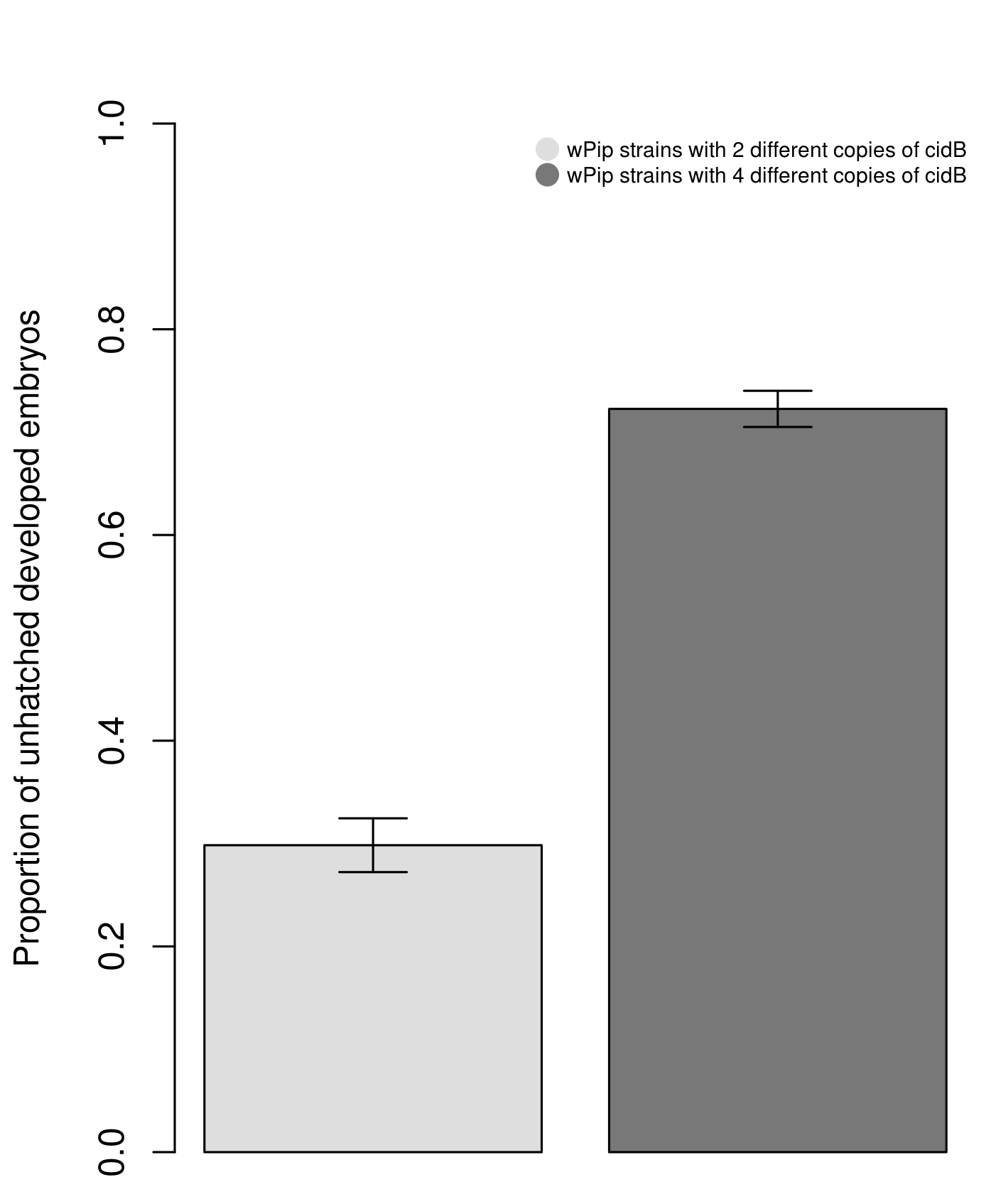

Supplement: S4 Fig — Lighter gray bar plot accounts for the 10 crosses involving males from Lavar and Utique lines hosting wPip, which harbor 2 different variants of cidB in their genomes, while darker gray bar plot accounts for the 10 crosses involving males from Tunis and Slab lines, both infected with wPip strains harboring 4 different variants of cidB. Error bars represent the standard error. The proportion of unhatched developed embryos was significantly higher for males hosting four-variants wPip strains than for males hosting two-variants wPip strains (Wilcoxon, W = 1159, p<0.001). (TIF) [file ppat.1007364.s010.tif]

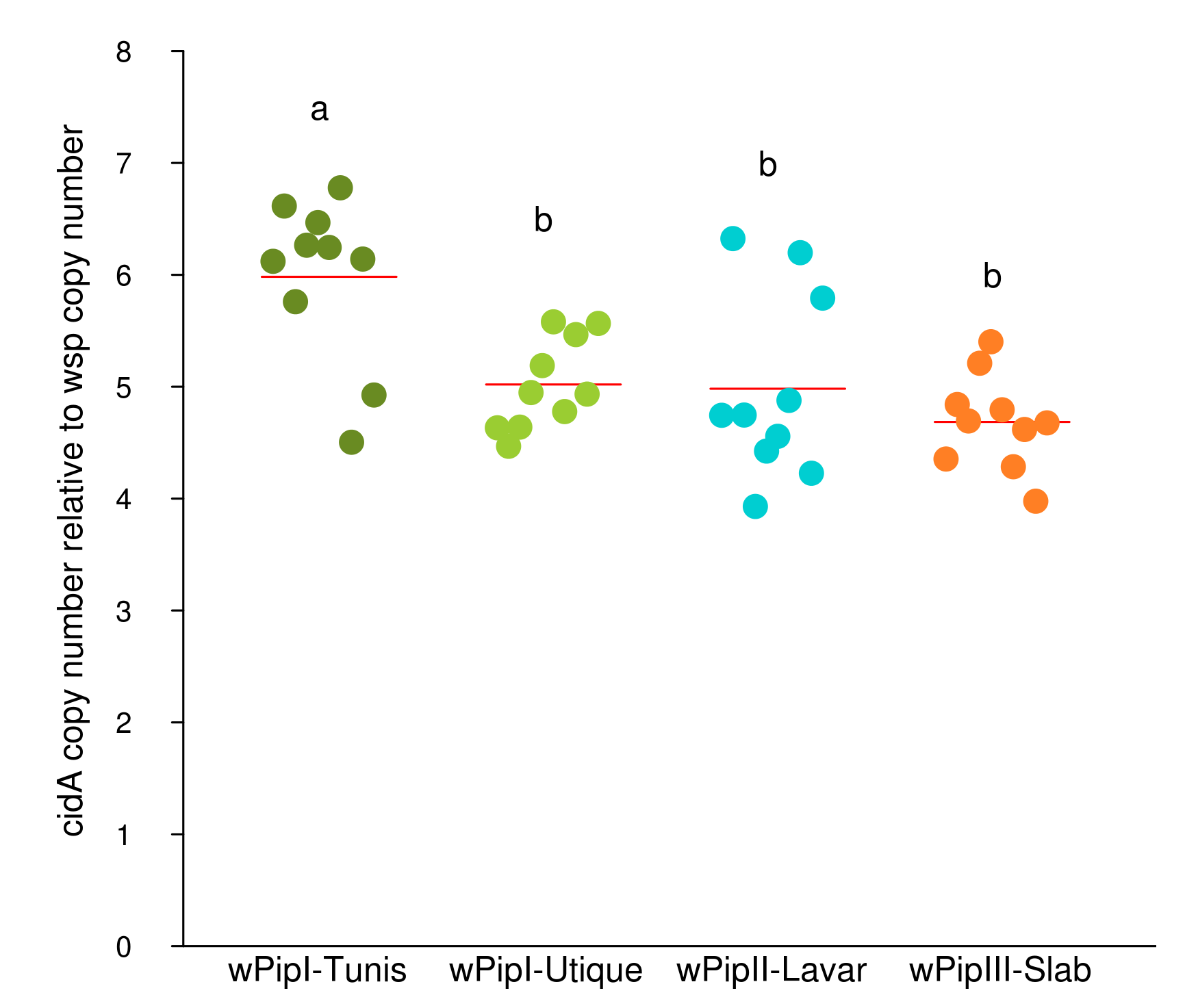

Supplement: S5 Fig — cidA copy number was measured by quantitative PCR as the ratio between the number of copies of the Wolbachia cidA gene and the Wolbachia wsp gene. The colored dots represent the cidA copy number per wPip genome in a male and the red strips represent the average cidA copy number per wPip genome for ten males per Mal lines. Letters represent the different statistical groups (i.e. means with the same letter are not significantly different). (TIF) [file ppat.1007364.s011.tif]

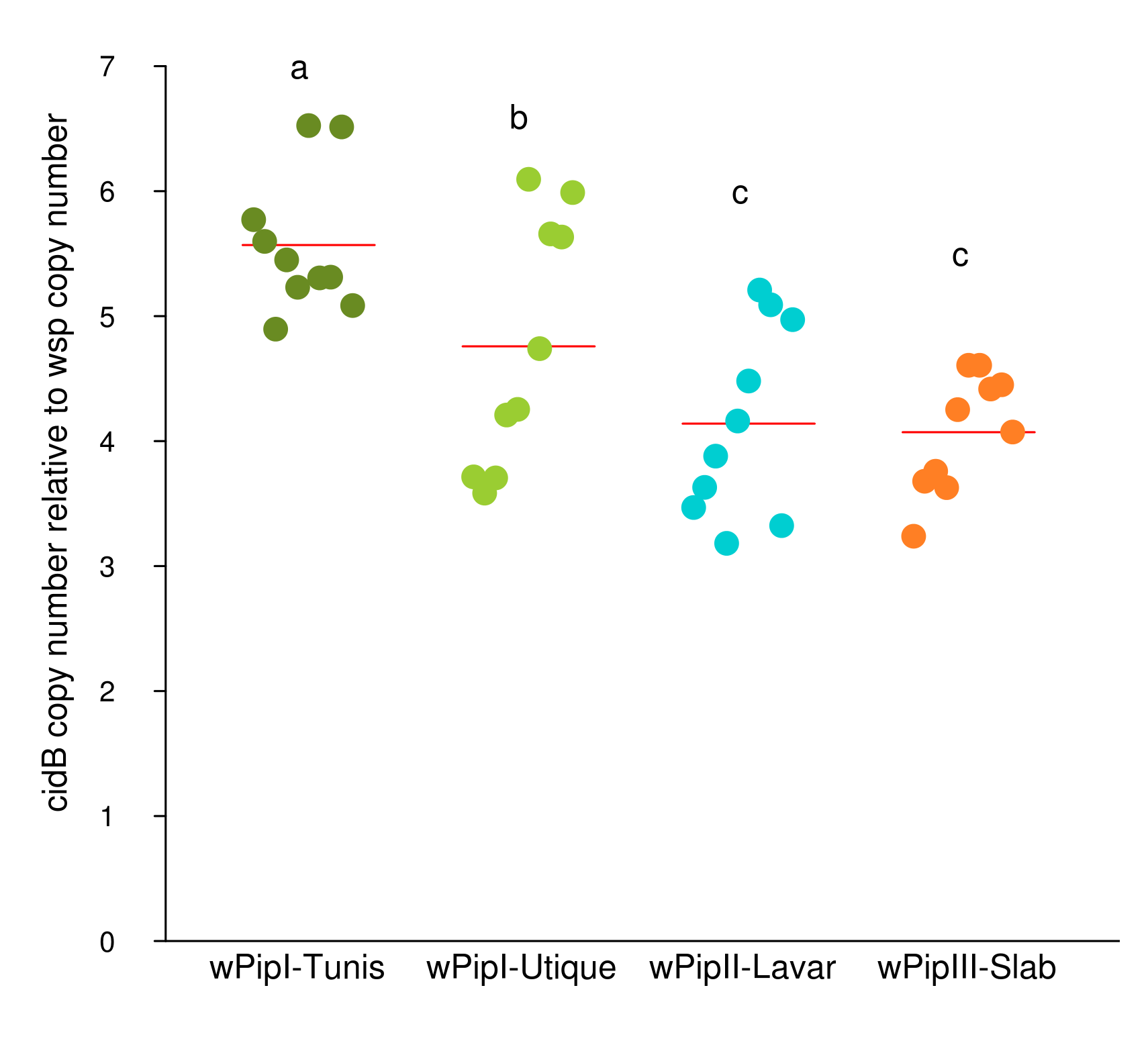

Supplement: S6 Fig — cidB copy number was measured by quantitative PCR as the ratio between the number of copies of the Wolbachia cidB gene and the Wolbachia wsp gene. The colored dots represent the cidB copy number per wPip genome in a male and the red strips represent the average cidB copy number per wPip genome for ten males per Mal lines. Letters represent the different statistical groups (i.e. means with the same letter are not significantly different). (TIF) [file ppat.1007364.s012.tif]

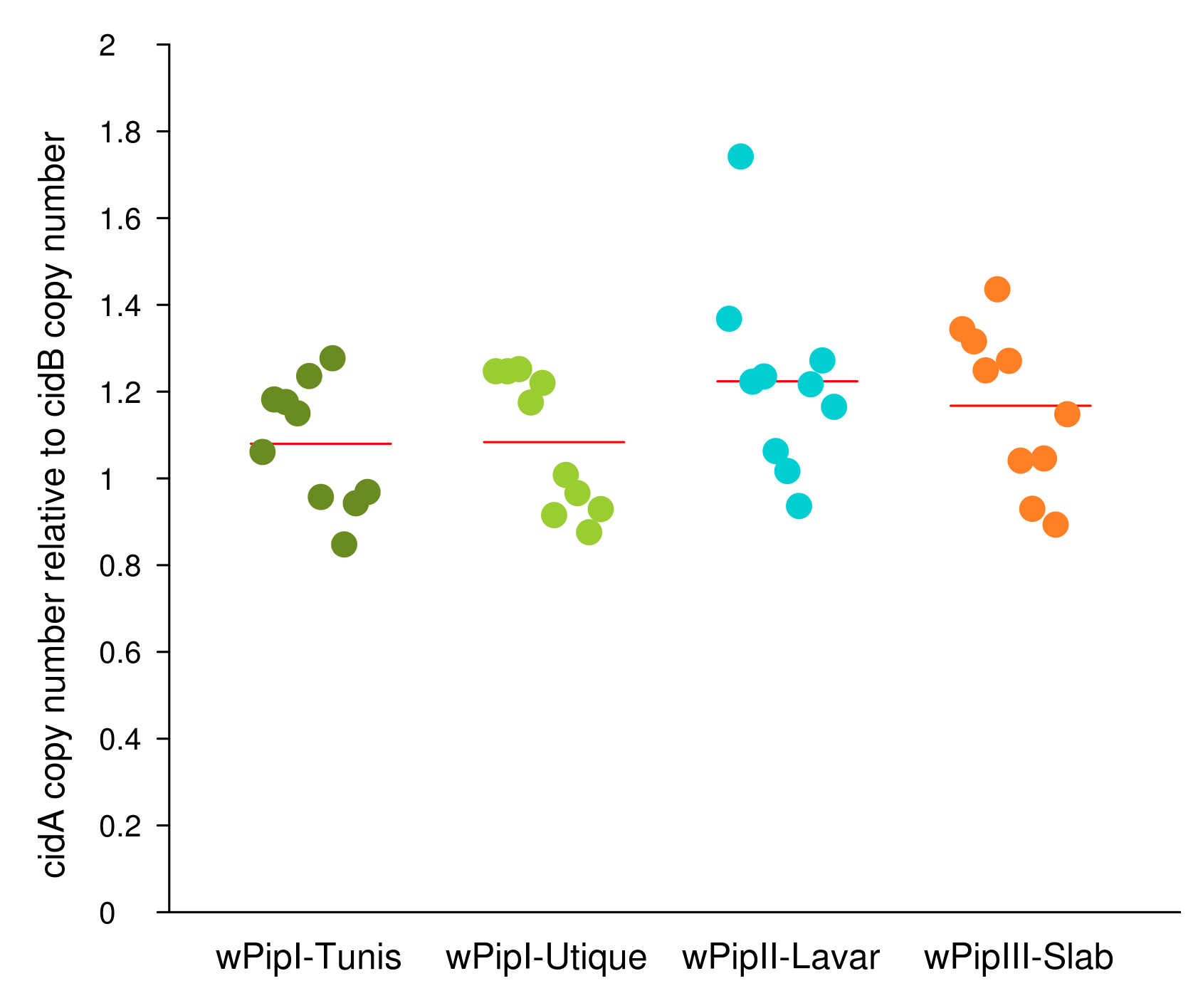

Supplement: S7 Fig — cidA/cidB copy number was measured by quantitative PCR as the ratio between the number of copies of the Wolbachia cidA gene and the Wolbachia cidB gene. The colored dots represent the cidA/cidB copy number per wPip genome in a male and the red strips represent the average cidA/cidB copy number per wPip genome for ten males per Mal lines. cidA/cidB copy number were not significantly different between the four wPip strains infecting the four Mal lines. (TIFF) [file ppat.1007364.s013.tiff]

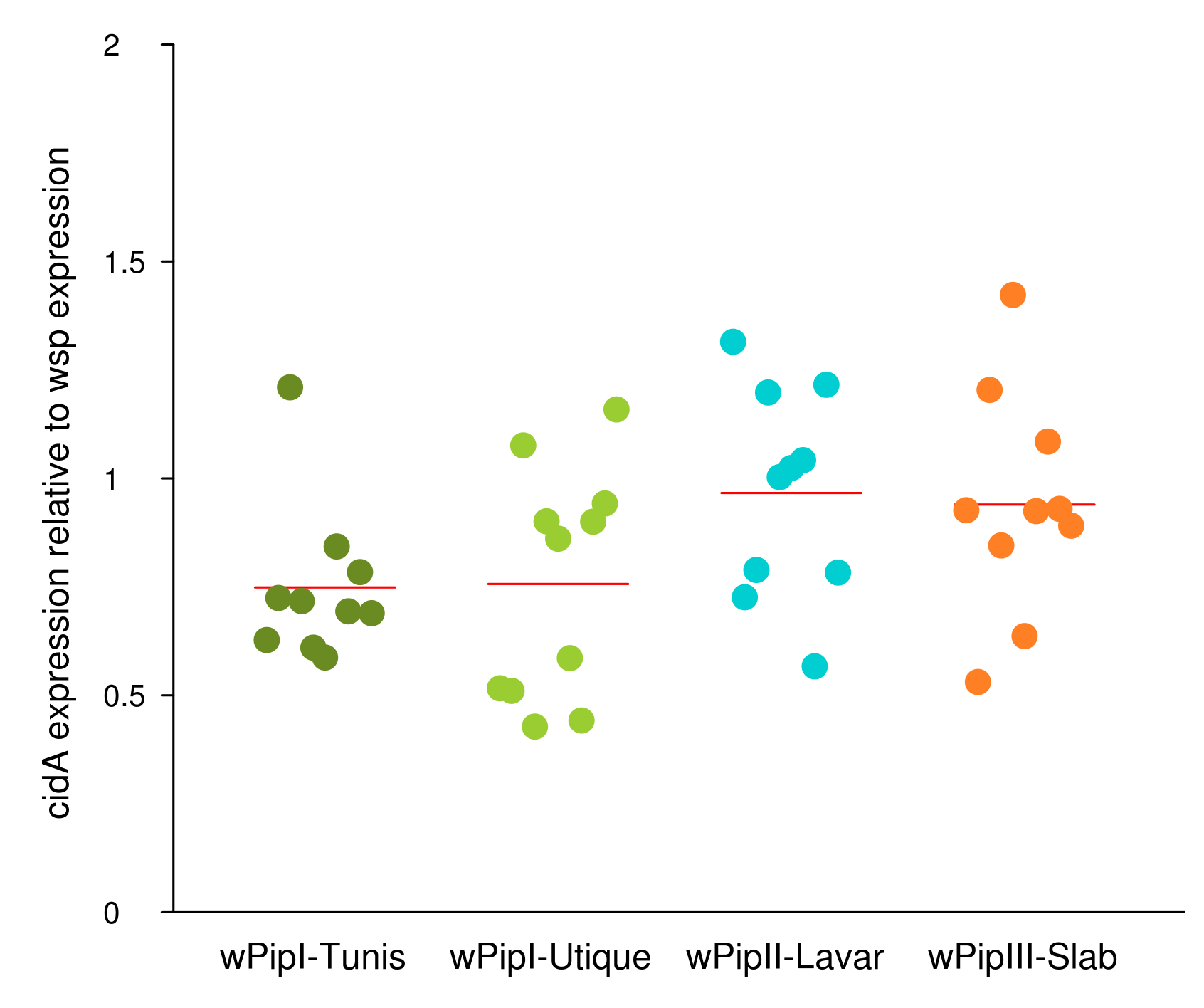

Supplement: S8 Fig — cidA expression was measured by quantitative PCR as the ratio between the Wolbachia cidA gene expression and the Wolbachia wsp gene expression. The colored dots represent the cidA expression level per wPip genome in a male and the red strips represent the average cidA expression level per wPip genome for ten males per Mal lines. Expression levels of cidA genes were not significantly different between the four wPip strains infecting the four Mal lines. (TIFF) [file ppat.1007364.s014.tiff]

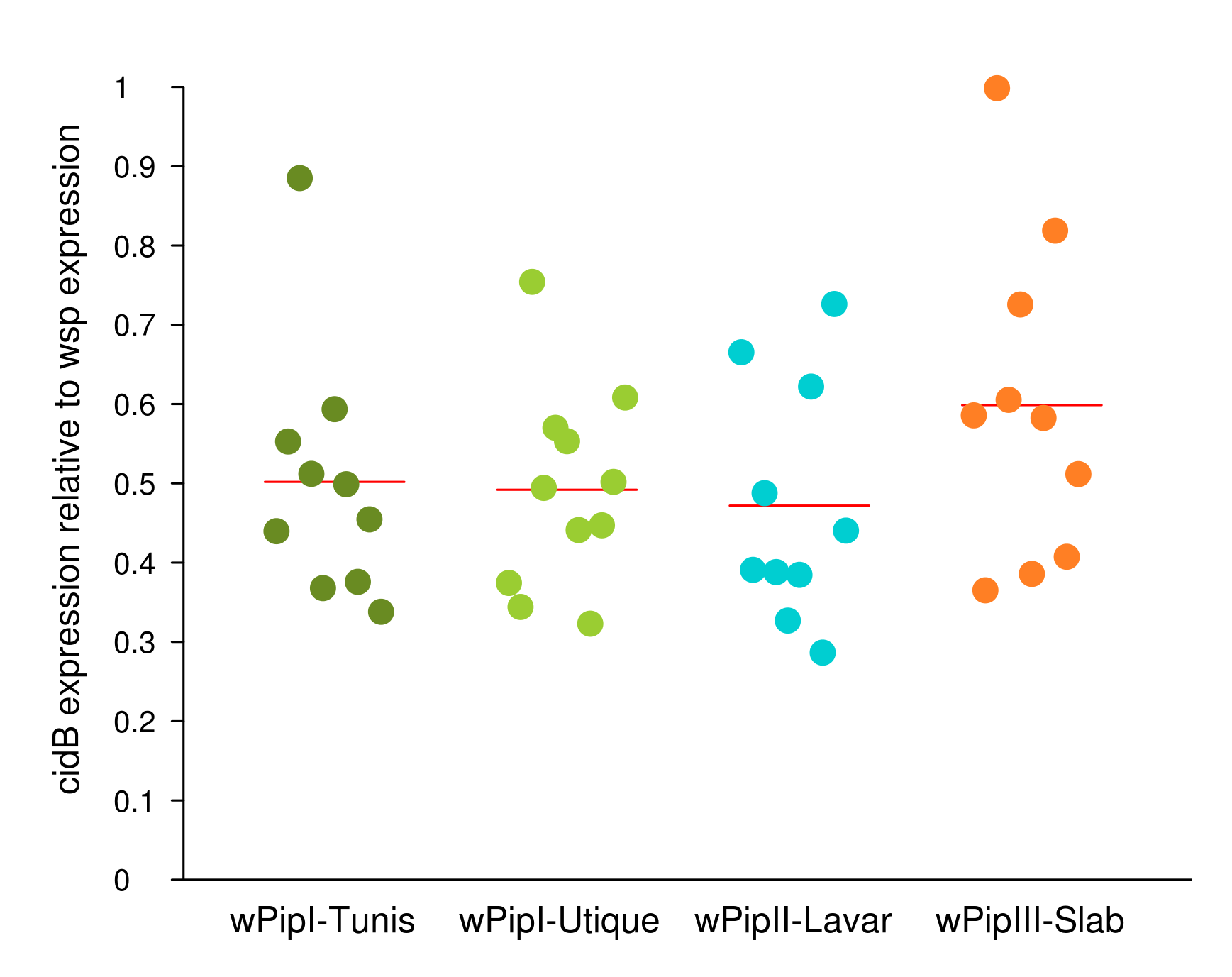

Supplement: S9 Fig — cidB expression was measured by quantitative PCR as the ratio between the Wolbachia cidB gene expression and the Wolbachia wsp gene expression. The colored dots represent the cidB expression level per wPip genome in a male and the red strips represent the average cidB expression level per wPip genome for ten males per Mal lines. Expression levels of cidB genes were not significantly different between the four wPip strains infecting the four Mal lines. (TIFF) [file ppat.1007364.s015.tiff]

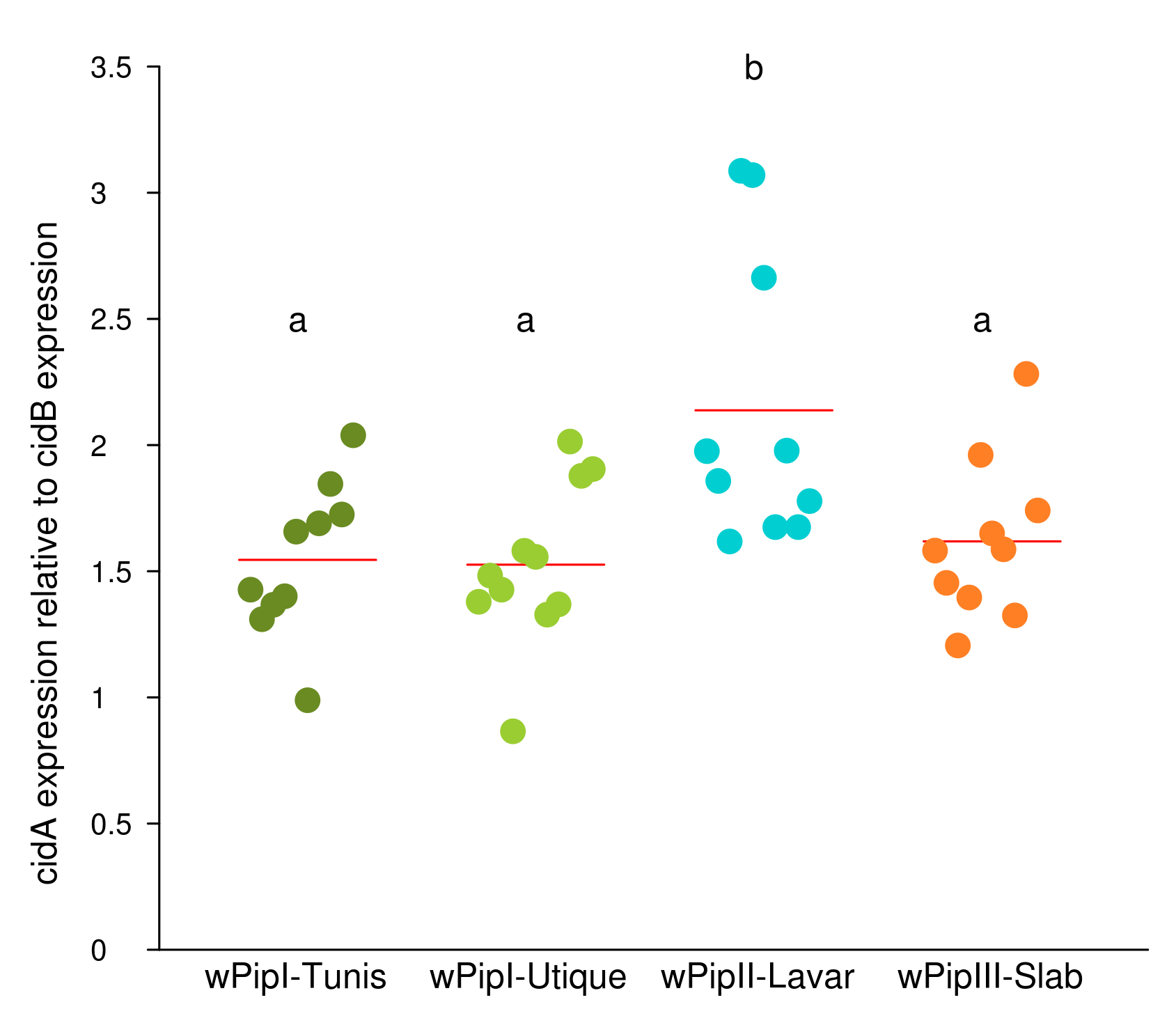

Supplement: S10 Fig — cidA/cidB expression levels was measured by quantitative PCR as the ratio between the number of copies of the Wolbachia cidA gene and the Wolbachia cidB gene. The colored dots represent the cidA/cidB expression level per wPip genome in a male and the red strips represent the average cidA/cidB expression per wPip genome for ten males per Mal lines. Letters represent the different statistical groups (i.e. means with the same letter are not significantly different). (TIFF) [file ppat.1007364.s016.tiff]
